# UV-B Radiation Triggers Phenolic Accumulation and Oxidative Stress Response in Lamiaceae Species: From Plant Defense to Green Dye Remediation

**DOI:** 10.3390/plants15142210

**Published:** 2026-07-20

**Authors:** Inês Mansinhos, Sandra Gonçalves, João Brás, Raquel Rodríguez-Solana, María José Aliaño Gonzalez, Bruno Medronho, Gema Pereira-Caro, José Manuel Moreno-Rojas, Anabela Romano

**Affiliations:** 1 MED—Mediterranean Institute for Agriculture, Environment and Development & CHANGE—Global Change and Sustainability Institute, Faculdade de Ciências e Tecnologia, Universidade do Algarve, Campus de Gambelas, 8005-139 Faro, Portugal; ifmansinhos@ualg.pt (I.M.); jtbras@hotmail.com (J.B.); raquel.rodriguez.solana@juntadeandalucia.es (R.R.-S.); mariajose.aliano@gm.uca.es (M.J.A.G.); bfmedronho@ualg.pt (B.M.); aromano@ualg.pt (A.R.); 2 Department of Agroindustry and Food Quality, Andalusian Institute of Agricultural and Fisheries Research and Training (IFAPA), Rancho de la Merced Center, Carretera Cañada de la Loba (CA-3102) Km 3.1., SN, 11471 Jerez de la Frontera, Cádiz, Spain; 3 Analytical Chemistry Department, University of Cádiz, 11510 Puerto Real, Cádiz, Spain; 4 Surface and Colloid Engineering, FSCN Research Center, Mid Sweden University, SE-851 70 Sundsvall, Sweden; 5 Department of Agroindustry and Food Quality, Andalusian Institute of Agricultural and Fisheries Research and Training (IFAPA), Alameda del Obispo Center, Avenida Menendez-Pidal, SN, 14004 Córdoba, Córdoba, Spain; mariag.pereira@juntadeandalucia.es (G.P.-C.); josem.moreno.rojas@juntadeandalucia.es (J.M.M.-R.); 6 Foods for Health Group, Instituto Maimónides de Investigación Biomédica de Córdoba (IMIBIC), Avenida Menendez-Pidal, SN, 14004 Córdoba, Córdoba, Spain

**Keywords:** UV-B induced stress, Mediterranean plants, phenolic extracts, HPLC-HRMS, Natural Deep Eutectic Solvents, BBD-RSM, cationic dye

## Abstract

The present study explores the impact of UV-B radiation on biochemical traits and phenolic profile of in vitro cultures (IC) and micropropagated plants (MP) of *Lavandula viridis* L’Hér and *Thymus lotocephalus* G. López and R. Morales. Two UV-B treatments were applied: a single 4 h exposure (UV-B 1) and repeated exposure over four consecutive days (UV-B 4). Additionally, the potential of phenolic-rich extracts loaded into alginate-based hydrogels for dye removal was also evaluated. UV-B exposure triggered oxidative stress in both species, particularly in MP, increasing hydrogen peroxide levels and lipid peroxidation, and affecting chlorophyll and carotenoid content. Both species responded by accumulating soluble sugars and phenolic compounds as a defense mechanism. Rosmarinic acid, the predominant phenolic compound, increased significantly under UV-B radiation in IC and MP. IC showed higher concentrations after UV-B 1 exposure, with *L. viridis* reaching 50.1 mg/g and *T. lotocephalus* 32.3 mg/g, increases of 16% and 41%, respectively, over the control. Polyphenol-loaded hydrogels showed high methylene blue adsorption efficiency, highlighting their potential as eco-friendly materials for wastewater treatment and environmental remediation. Optimal adsorption conditions were determined using Box–Behnken design and Response Surface Methodology, demonstrating the applicability of these natural hydrogels as sustainable and efficient materials for dye removal from contaminated wastewater.

## 1. Introduction

Water contamination represents one of the most significant global challenges, primarily due to the textile industry’s inadequate wastewater disposal practices. Annually, the global production of synthetic dyes exceeds 700,000 tons [1], with the textile industry alone accounting for 10,000 tons [2]. Many of these dyes have been identified as toxic, carcinogenic, mutagenic, and teratogenic, posing considerable ecotoxicological risks to both human health and aquatic organisms [3]. Among the numerous synthetic dyes, methylene blue (MB), is widely used in various industries (especially textiles) and is responsible for ca. 20% of global wastewater contamination [3]. To mitigate this environmental issue, a range of chemical and physical treatment methods have been developed, including flotation, coagulation/flocculation, chemical oxidation, irradiation, solvent extraction, precipitation, and membrane-based separation [4]. However, these traditional methods have significant drawbacks, including high costs, hazardous byproducts, sludge formation, heavy metal production, and operational inefficiencies [4]. Consequently, there has been a growing interest in eco-friendly and cost-effective alternatives, such as adsorption, as a promising solution for dye removal from contaminated wastewater [1]. At the core of this process is a surface phenomenon in which molecules, atoms, or ions from a gas, liquid, or dissolved solid (adsorbate) adhere to the surface of a substance (adsorbent), facilitating pollutant removal [4].

Plant-derived products from agricultural biomass and waste materials have demonstrated considerable potential as bioadsorbents, particularly in the context of dye removal [5,6]. The majority of reports consider agricultural biomass in the form of raw, modified, or carbonaceous materials (biochar or activated carbon) for the removal of harmful aquatic contaminants [7]. However, carbonaceous adsorbents are expensive, exhibit batch-to-batch variability [8], and are vulnerable to erosion and disintegration in water flow, potentially leaching harmful pollutants into the environment [9].

Plants contain high concentrations of biological compounds, particularly phenolics, which feature functional groups such as hydroxyl (–OH) and carboxyl (–COOH) that serve as active binding sites for biosorption [10]. Due to the complexity of plant matrices, the use of plant extracts instead of raw materials offers several advantages when phytochemicals (e.g., phenolics) are the desired compounds. These extracts have gained attention in the development of innovative adsorbents, such as hydrogels, for pollutant removal. The functional groups present in phenolic compounds exhibit excellent properties, including reduction, chelation, and capping of metal or metal oxide nanomaterials [11,12], further enhancing the performance of the bioadsorbents [13]. Lignin, for instance, which possesses a complex structure with phenolic compounds, is a subject of extensive research due to its effectiveness in removing dyes from dyeing wastewater because of its abundant hydroxyl groups, which confer high hydrophilicity and affinity for cationic dyes like MB [14]. A recent study has demonstrated the efficacy of an eco-friendly adsorbent synthesized through grafting gallic and caffeic acids onto a chitosan substrate. This novel adsorbent has been shown to be highly effective in the removal of cationic dye MB from aqueous solutions [15].

A strategy employed to enhance the production of secondary metabolites in plants is their exposure to abiotic stress. Previous studies have demonstrated that abiotic factors such as drought [16], temperature [17], and nutrient stress [18] affect the phenolic profile and related biological properties in *Lavandula viridis* L’Hér and *Thymus lotocephalus* G. López and R. Morales, two Lamiaceae aromatic species rich in phenolic compounds, especially phenolic acids like rosmarinic, caffeic, and salvianolic acids [19]. Notwithstanding the considerable number of phenolic compounds present in aromatic and medicinal plants, their potential for the development of bioadsorbents remains largely unexplored. It is noteworthy that research on *Thymus numidicus* [20] and *Lavandin* [21] has demonstrated their capacity to produce valuable compounds for the removal of MB from water.

In the present study, it was hypothesized that UV-B radiation could induce oxidative stress in *L. viridis* and *T. lotocephalus*, leading to an increased production of nonenzymatic antioxidants, specifically phenolic compounds, and consequently affecting the MB removal capacity of hydrogels loaded with plant extracts. To test this hypothesis, high-performance liquid chromatography–high-resolution mass spectrometry (HPLC–HRMS)-based phenolic metabolite profiling was performed, combined with oxidative stress analyses in in vitro cultures (IC) and micropropagated plants (MP) of *L. viridis* and *T. lotocephalus* exposed to two distinct UV-B treatments. The specific objectives of this study were: (a) to determine whether UV-B radiation influences the production of phenolics in *L. viridis* and *T. lotocephalus* IC and MP; (b) to assess the efficacy of phenolic-rich plant extracts incorporated into hydrogels for MB removal from aqueous solutions; and (c) to identify specific phenolic compounds that may contribute to enhanced MB adsorption. To the best of our knowledge, this is the first report studying the impact of UV-B radiation on *L. viridis* and *T. lotocephalus* and utilizing Natural Deep Eutectic Solvent-based extracts as a sustainable and novel bioadsorbent for the removal of MB from water.

## 2. Results and Discussion

### 2.1. Impact of UV-B Radiation on Photosynthetic Pigments, Oxidative Stress Biomarkers, and Soluble Sugars

Among the various UV-B targets, the primary one is the photosynthetic apparatus [22]. The impact of UV-B on plant photosynthesis has been observed at various levels, including changes in leaf structure and morphology [23], as well as in the photosynthetic pigment apparatus, specifically in Calvin cycle enzymes and in membranes involved in the energy transduction system [24]. However, it should be noted that these effects depend on several factors, including the intensity and duration of UV-B exposure, as well as the plant species [25]. While UV-B radiation has been observed to induce protective mechanisms [26,27], the detrimental effects on photosynthetic pigments are significant, highlighting the challenges that plants face in adapting to increased UV exposure.

**Figure 1:**
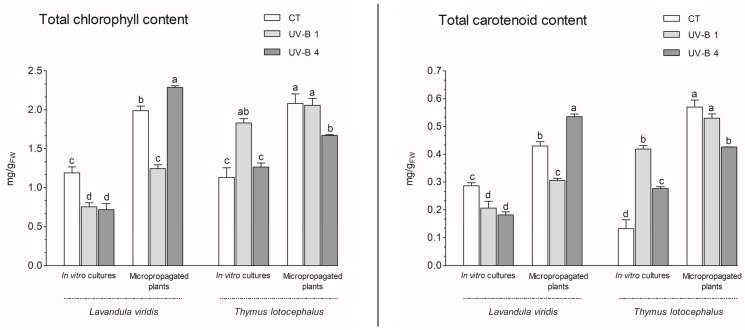
Effect of two distinct UV-B treatments (UV-B 1 and UV-B 4) on the total chlorophyll and carotenoid contents of in vitro cultures and micropropagated plants of *Lavandula viridis* and *Thymus lotocephalus*. For each species, results were analyzed using one-way analysis of variance (ANOVA). Graph bars followed by different letters (a–d) are significantly different at *p* < 0.05 (Duncan’s New Multiple Range Test).

**Figure 2:**
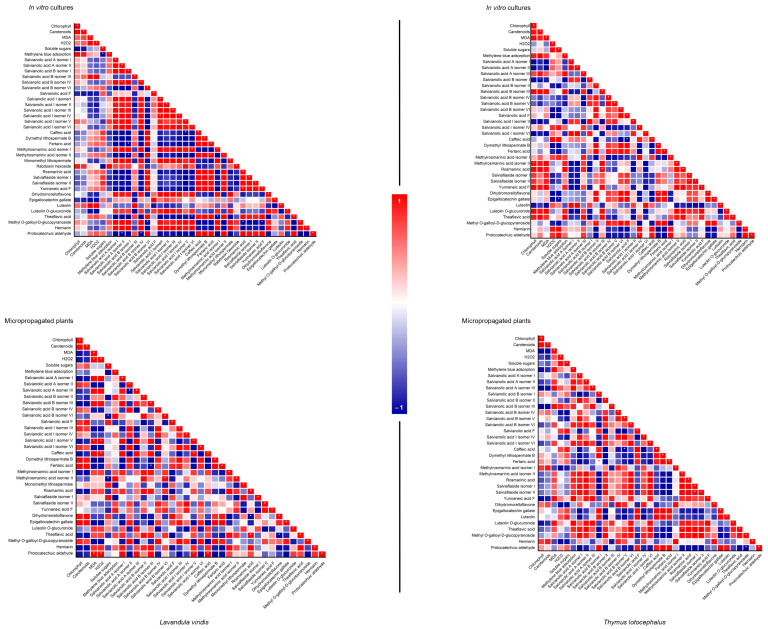
Heatmap corresponding to Pearson’s correlation between the contents of chlorophylls, carotenoids, hydrogen peroxide (H_2_O_2_), lipid peroxidation (MDA), soluble sugars, methylene blue adsorption, and the individual phenolic compounds identified by HPLC-HRMS in extracts from in vitro cultures and micropropagated plants of *Lavandula viridis* and *Thymus lotocephalus* subjected to two distinct UV-B treatments (UV-B 1 and UV-B 4). * Correlation is significant (*p* ≤ 0.01).

The UV-B treatment (UV-B 1 and UV-B 4) significantly affected the leaf pigments in both species and at both plant micropropagation stages (IC and MP) (Figure 1). The shorter exposure period had a detrimental effect on photosynthetic pigments in *L. viridis* (−37% total chlorophylls and −28 to 29% carotenoids in both IC and MP). Numerous studies have demonstrated that UV-B radiation leads to a decrease in chlorophyll levels across various plant species from the Lamiaceae family, such as *Thymus vulgaris* L. [28], *Ocimum basilicum* L. [29], *Prunella vulgaris* L. [30], and *Mentha aquatica* L. [31], as well as from other families [25,26,32,33]. In contrast, *T. lotocephalus* exhibits an increase in both pigments (+62% total chlorophylls and +217% carotenoids in IC), possibly due to phenolic compound protection. In agreement with previous studies [26], a strong correlation was identified between chlorophyll/carotenoid levels and several phenolic compounds present in *T. lotocephalus* IC (Figure 2). When the UV-B treatment was repeated (UV-B 4), the response varied depending on the plant species and micropropagation stage. Similar contradictory effects have been reported in other species [29,34].

UV-B radiation is known to enhance the generation of reactive oxygen species (ROS) in plants by modifying electron transport systems (ETS) and diverting electron paths in photosynthetic and respiratory systems [24]. The disruption of ETS in diverse cellular compartments leads to increased formation and accumulation of ROS, such as hydrogen peroxide (H_2_O_2_) [24]. Insufficient regulation of H_2_O_2_ by the plant’s antioxidant system can result in damage to biological macromolecules, including lipids, proteins, and DNA [22]. Specifically, high concentrations of H_2_O_2_ have been observed to cause harm to cellular membranes by producing hydroxyl radicals (OH), thereby promoting lipid peroxidation [18]. This is supported by the robust positive correlation observed in all cases (except *T. lotocephalus* IC) between the H_2_O_2_ levels and MDA, a widely used indicator of lipid peroxidation (Figure 2). The findings of this study demonstrate that UV-B radiation increases the levels of H_2_O_2_ and MDA, both well-established markers of oxidative stress, in MP of both species, although the extent of this increase varies depending on the specific UV-B treatment (Table 1).

In contrast, neither UV-B treatment increased H_2_O_2_ accumulation in the IC of either species. Nevertheless, a significant increase in lipid peroxidation was observed in *T. lotocephalus* following a single UV-B application (UV-B 1). The low H_2_O_2_ levels suggest that UV-B exposure did not trigger pronounced photo-oxidative stress in the IC, possibly reflecting the activation of efficient antioxidant and repair mechanisms that limited ROS accumulation. The contrasting responses observed between IC and MP are likely related to differences in developmental stage and growth conditions. The IC consisted of 7-week-old shoots maintained under controlled in vitro conditions, whereas the MP had been acclimatized and grown under ex vitro conditions for 16 months before UV-B exposure. These differences in physiological maturity and environmental acclimation may have influenced antioxidant capacity and stress-response mechanisms, contributing to the distinct oxidative responses observed. In other plants, UV-B-induced damage was linked to increased levels of both H_2_O_2_ and MDA [26,31,35,36]. For example, in *P. vulgaris*, no significant rise in MDA levels was observed after UV-B exposure, suggesting strong resilience to this radiation [30]. In this study, a notable difference in ROS levels was observed between IC and MP in *T. lotocephalus*, with IC accumulating 54–86% less H_2_O_2_ than MP. A similar trend was previously reported for this species under temperature stress [17]. In the case of *L. viridis*, however, this difference was not significant, possibly due to the higher total phenolic content produced by IC (see Section 2.2 for further discussion). Higher phenolic levels have been associated with enhanced H_2_O_2_ scavenging [22]. This assumption is further supported by the strong negative correlations observed in *L. viridis* IC between H_2_O_2_ levels and specific phenolic compounds (Figure 2). In contrast, the lower levels of oxidative stress markers and the improved photosynthetic pigment content observed in *L. viridis* MP following repeated UV-B exposure (UV-B 4) suggest the development of an acclimation response. The greater physiological maturity of these plants, acquired during 16 months of ex vitro growth, may have favored the establishment of more efficient antioxidant and photoprotective mechanisms, enabling tighter regulation of UV-B-induced oxidative stress and better preservation of photosynthetic function.

The accumulation of soluble sugars in response to UV-B stress may serve as a defensive mechanism in plants. Sugars can function as osmolytes, helping to maintain cellular integrity [17], and also contribute to the synthesis of phenolic compound precursors [37]. In the present study, both UV-B treatments significantly affected the soluble sugar concentrations in IC and MP of both species, with levels consistently higher than those observed in the respective controls (Table 1). Numerous studies support these findings, showing that plants accumulate excess sugars as a defensive mechanism against UV-B stress. For instance, Hamid et al. [38] observed that UV-B exposure increased the soluble sugar content in both roots and leaves of white clover during the vegetative stage. Sucrose, glucose, and fructose are among the key soluble sugars involved in cellular metabolism, plant structure regulation, and protection against oxidative damage [35]. Their concentrations have been shown to increase in response to UV-B radiation in several plant species [35,39,40].

### 2.2. Phenolic Profile Analysis by HPLC-HRMS

Phenolic compounds play a central role in protecting plants against damage caused by abiotic stress, particularly by scavenging ROS [19]. Our previous studies demonstrated that abiotic stressors such as drought [16], temperature (low/high) [17], and nutrient deficiency [18] affect the accumulation of phenolic compounds (along with their biological properties) in *L. viridis* and *T. lotocephalus*.

**Figure 3:**
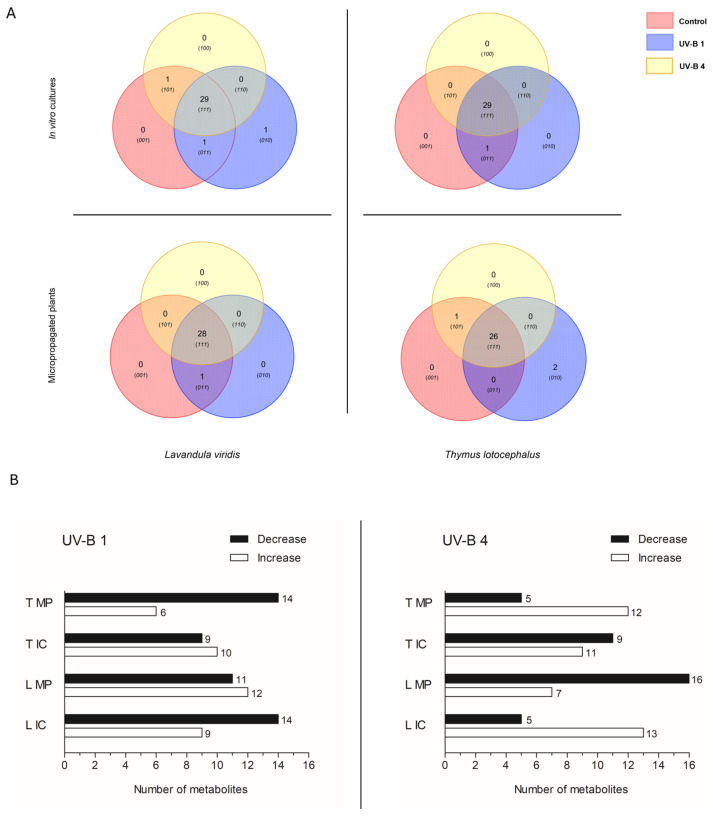
Overview of significantly changed metabolites in the extracts from in vitro cultures and micropropagated plants of *Lavandula viridis* and *Thymus lotocephalus* in response to two distinct UV-B treatments (UV-B 1 and UV-B 4). (**A**). Venn Diagram showing the number of quantified phenolic compounds. (**B**). Total significantly increased and decreased (*p* < 0.05) metabolites under UV-B 1 and UV-B 4 treatments. L: *Lavandula viridis*; T: *Thymus lotocephalus*; IC: in vitro cultures; MP: micropropagated plants.

In the present study, the effects of UV-B radiation on the phenolic profiles of *L. viridis* and *T. lotocephalus* were investigated. A total of 34 phenolic compounds were identified in IC and 32 in MP of *L. viridis,* of which 32 (in IC) and 29 (in MP) were successfully quantified. In *T. lotocephalus*, 31 phenolics were identified, with 30 quantified in IC and 29 in MP (Table 2 and Figure 3A). Phenolic acids were the most abundant class of compounds, accounting for 77% and 73% of the total phenolics quantified in *L. viridis* and *T. lotocephalus*, respectively. Furthermore, five flavonoids, a coumarin derivative, and a hydroxybenzaldehyde were also quantified in both species. A significant variation in the total phenolic contents was observed when comparing the results obtained by HPLC-HRMS for both species after exposure to different UV-B radiation treatments.

**Figure 4:**
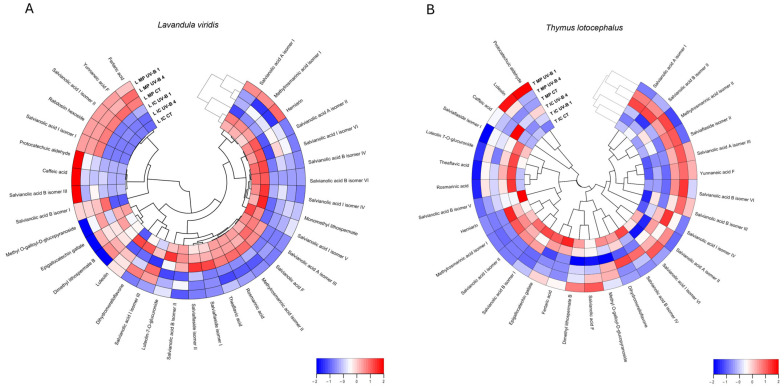
Polar heatmap (Pearson’s correlation) with a dendrogram representing the clustering of similarity (or distance) among plant extracts and individual phenolic compounds (identified by HPLC-HRMS) produced by in vitro cultures and micropropagated plants of *Lavandula viridis* (**A**) and *Thymus lotocephalus* (**B**) subjected to two distinct UV-B treatments (UV-B 1 and UV-B 4). L: *Lavandula viridis*; T: *Thymus lotocephalus*; IC: in vitro cultures; MP: micropropagated plants.

The total phenolic content of *L. viridis* ranged from 42.5 to 81.6 mg/g_DW_, while in *T. lotocephalus*, it varied from 35.9 to 50.8 mg/g_DW_. The highest values were achieved in both cases in IC. The difference between IC and MP was particularly pronounced in *L. viridis,* with increases in total phenolics of 56, 68 and 92% in the CT, UV-B 1, and UV-B 4, respectively (Table 2 and Figure 4A). These results demonstrate that elicitation of IC with UV-B radiation is an effective strategy for enhancing phenolic compound production in this species. Similarly, Pandey and Pandey-Rai [32] reported comparable results after stimulating in vitro cultures of *Artemisia annua* L. with UV-B radiation, observing a significant induction of 92% in total phenolic compounds after just 3 h of UV-B exposure. IC have been shown to be a powerful approach for studying plant responses to abiotic stress, given their ability to alter growth conditions (e.g., light, temperature, and media composition). Additionally, they enable large-scale, homogeneous plant material production under controlled, pathogen-free conditions, while also supporting species conservation [16]. The findings obtained emphasize the pivotal role of phenolic compounds in enabling plants to withstand UV-B stress, in agreement with previous reports on other plant species, including *M. aquatica* [31], *A. annua* [32], *P. vulgaris* [30], *Cajanus cajan* L. [26], *Lactuca sativa* L. [35], *Olea europaea* L. [41], and *Solanum lycopersicum* L. [36].

The individual effects (in each metabolite) were highly variable and dependent on the plant species and plant material. The UV-B 1 treatment had a more adverse effect on both species than UV-B 4, leading to a decline in the production of 23 metabolites in *T. lotocephalus* and 25 metabolites in *L. viridis* (Figure 3B). The hierarchical cluster analysis data, displayed as a polar heatmap with a dendrogram (Figure 4A,B), indicate that, based on the phenolic profile, samples from both species were grouped according to their micropropagation stage, with greater similarity observed within IC samples and within MP samples. Rosmarinic acid is the most abundant phenolic compound of both species investigated, accounting for 53–67% (IC) and 37–52% (MP) of the total phenolic compounds in *L. viridis*, and 53–64% (IC) and 48–55% (MP) in *T. lotocephalus* (Table 2). The investigation revealed that UV-B treatment significantly enhanced the biosynthesis of total phenolic acids in both species, with rosmarinic acid demonstrating a particularly notable response (Table 2). The highest content of this compound was observed in the IC of both *L. viridis* (50.1 ± 0.1 mg/g_DW_) and *T. lotocephalus* (32.3 ± 0.0 mg/g_DW_) after exposure to UV-B 1 treatment (Table 2 and Figure 4A,B). UV-B radiation has also been reported to promote the accumulation of this phenolic acid in the callus of *Mirabilis himalaica* [42], and in plants such as *Perilla frutescens* L. [43], *P. vulgaris* [30,44] and *O. basilicum* [29]. It is interesting to note that, after two weeks of UV-B exposure, the content of rosmarinic acid decreased in calli and shoots of *Echium orientale* L. harvested immediately but increased in those harvested one week later [45]. Once again, in vitro culture proved to be a very useful approach for producing phenolic compounds. In *L. viridis*, the discrepancy between the IC and the MP for the synthesis of rosmarinic acid was pronounced, with this compound being produced at a rate that was twice as high in all cases (CT, UV-B 1, and UV-B 4) (Table 2 and Figure 4A).

Rosmarinic acid has been widely studied due to its biological activities. Its therapeutic potential spans various health conditions, including cancer, neurodegeneration, and diabetes, due to its anti-inflammatory and antioxidant properties [46]. Additionally, rosmarinic acid has been identified as a promising candidate for protecting the skin from UV-B-induced oxidative damage by enhancing antioxidant enzyme expression and activating signaling pathways [47], as well as preventing mitochondrial dysfunction [48]. These mechanisms help mitigate oxidative stress and preserve cell viability. The relationship between rosmarinic acid and UV-B radiation is bidirectional; while rosmarinic acid protects against UV-B-induced damage in cells, UV-B radiation also stimulates its production in plants, as observed in this study.

As previously reported [16,17,18], the galloyl ester methyl *O*-galloyl-D-glucopyranoside was also a major bioactive compound in extracts from both species. Notably, the synthesis of this compound remained unaffected by various abiotic stresses, including drought [16], temperature (high/low) [17], and nutrient limitation [18]. However, this was not the case for UV-B stress, which led to a decline in its production (−0.2 to 40.7% in *L. viridis* and −0.9 to 34.8% in *T. lotocephalus*) (Table 2 and Figure 4A). Conversely, the rosmarinic acid derivative dimethyl lithospermate B (another abundant compound in both species) exhibited a response only in the MP of *L. viridis*, with a 42% reduction after the UV-B 1 treatment.

Both *L. viridis* and *T. lotocephalus* are rich in various salvianolic acids (rosmarinic acid derivatives), particularly salvianolic acids A, B, F, and I. Notably, in IC, salvianolic acids accounted for 54% of the total quantified phenolic acids in *L. viridis* and 59% in *T. lotocephalus,* whereas in MP, this percentage was 57% for both species. In most cases (except *L. viridis* IC), the content of total salvianolic acids decreased after exposure to UV-B treatments (Table 2). In contrast, *Salvia miltiorrhiza* Bunge treated with UV-B exhibited higher levels of 28 metabolites, including 12 salvianolic acids [49].

In the present study, salvianolic acid B (isomer IV) (4.2–13.9 mg/g_DW_ in the IC and 4.6–6.8 mg/g_DW_ in the MP) and salvianolic acid A (isomer I) (0.5–0.7 mg/g_DW_ in the IC and 0.5–1.1 mg/g_DW_ in the MP) were the most abundant salvianolic acids in *L. viridis* and *T. lotocephalus*, respectively. The effect of UV-B treatment on salvianolic acid B (isomer IV) production in *L. viridis* varied depending on the plant material and the UV-B treatment (Table 2 and Figure 4A). Conversely, the production of salvianolic acid A (isomer I) considerably decreased after exposure to UV-B stress in both IC and MP of *T. lotocephalus* (Table 2 and Figure 4B). These findings suggest that UV-B radiation can be a highly effective stress factor to induce the production of specific phenolic acids. Specifically, the Venn diagram (Figure 4A) demonstrated that UV-B 1 treatment stimulated the biosynthesis of one compound in *L. viridis* IC [salvianolic acid B (isomer VI)] and two compounds in *T. lotocephalus* MP [salvianolic acid I (isomer II) and fertaric acid], which were absent in their respective controls (Table 2). These findings suggest that UV-B radiation is an effective elicitor of the accumulation of specific phenolic acids. Although the response was species- and metabolite-dependent, repeated UV-B exposure (UV-B 4) promoted the accumulation of several salvianolic acid derivatives, including salvianolic acid A isomer I, salvianolic B isomer IV, salvianolic acid F and salvianolic acid I isomers II, III, IV and VI in *L. viridis* IC, as well as salvianolic acid A isomers II and III and salvianolic acid B isomers III and VI in *T. lotocephalus* MP. The enhanced accumulation of these compounds under prolonged UV-B exposure suggests that, together with rosmarinic acid, they may also contribute to the antioxidant defense system by mitigating UV-B-induced oxidative stress.

UV-B treatments also enhanced the total flavonoid content, aligning with previous reports [26,30,31,32,36]. Theaflavic acid was identified as the major flavonoid detected (0.6–1.2 mg/g_DW_), and its synthesis was significantly increased under UV-B stress (+5.3–26.5%). To the best of our knowledge, this is the first study reporting the positive effect of UV on the biosynthesis of this compound. Theaflavic acid, a type of theaflavin commonly isolated from black tea, has been reported to exhibit significant neuroprotective properties, particularly in ischemic stroke models [50,51].

### 2.3. Hydrogel as a Green Approach to MB Adsorption

#### 2.3.1. Point of Zero Charge (pH_PZC_) and FTIR Characterization

In this study, we evaluated, for the first time, the potential of SA beads incorporating green extracts from *L. viridis* and *T. lotocephalus* for the removal of dyes from aqueous solutions. To elucidate the charge characteristics of the hydrogels, their pH at the point of zero charge (pH_PZC_) was analyzed. This parameter represents the pH at which a material’s net charge is neutral, thereby influencing its interactions with ions and molecules in the solution. The pH_PZC_ measurements for hydrogels incorporating extracts from both species and micropropagated stages exhibited similar behavior, approximately at pH 4 (Figure S2). These findings confirm the acidic nature of the hydrogels, indicating that above ca. pH 4, all samples display a negative net charge, with the most pronounced negative charge observed at pH 10. It is noteworthy that such a pronounced negative charge density at pH 10 is particularly advantageous for the adsorption of cationic dyes, such as MB, as discussed in the subsequent section.

The interplay between pH and dye charge significantly influences the mechanism of MB adsorption, with the outcome contingent on the specific pH values. The adsorption process is attributed to distinct interactions between the negatively charged groups of phenolic compounds present in the extract and MB, a cationic dye. Phenolic compounds are characterized by at least one phenol unit, defined as a hydroxyl (–OH) group directly bonded to an aromatic hydrocarbon backbone [52]. The major phenolic compound found in both plant extracts, rosmarinic acid, contains two phenolic rings, each with two –OH groups. Under alkaline conditions, the likelihood of protonation of the –OH groups progressively decreases, increasing the negative charge of the phenolic compounds. Consequently, the adsorption process is predominantly driven by electrostatic interactions between the negatively charged phenolic groups and the cationic MB [53,54]. The most significant electrostatic interaction is anticipated to occur between the carboxylate group (–COO) of rosmarinic acid, which has a pKa of approximately 3.57 [55], and the protonated quaternary amine of MB. Additionally, the phenolic groups of rosmarinic acid can ionize under basic conditions, given that the pKa of phenol is 9.98 [56]. Therefore, electrostatic interactions may also occur between partially deprotonated –OH groups and MB. Conversely, if deprotonation is incomplete, hydrogen bonds may still form between these –OH groups and the non-protonated amines of MB. These interactions are supported by the ATR-FTIR results obtained in this study (Figure S3). Although no distinct shifts were observed in the characteristic C–N or C=N stretching bands of MB (typically between 1330 and 1640 cm^−1^), several bands within the 1000–1250 cm^−1^ region (attributed to C–O and C–O–C vibrations of phenolic compounds) were significantly diminished or disappeared upon bead formation. At low pH, the presence of high concentrations of H^+^ ions in solution retains the –OH groups in their non-ionized state. This occurs due to the competition between the H^+^ ions and the MB cations for available active adsorption sites on the phenolic compounds, thereby reducing the adsorption efficiency [57]. Consequently, within an acidic environment, the interaction between MB and phenolics is predominantly governed by hydrogen bonding between the amine groups of MB and the non-ionized –OH groups. According to the ATR-FTIR results (Figure S3), in the SA + MB system, a slight red shift and broadening of the O–H/N–H stretching band (~3250 cm^−1^) were observed, consistent with hydrogen bond formation between hydroxyl and amine groups. In the SA + extract system, a similar broadening (though without noticeable shifting) was observed, suggesting H-bonding also occurs between SA and the phenolic –OH groups.

**Figure 5:**
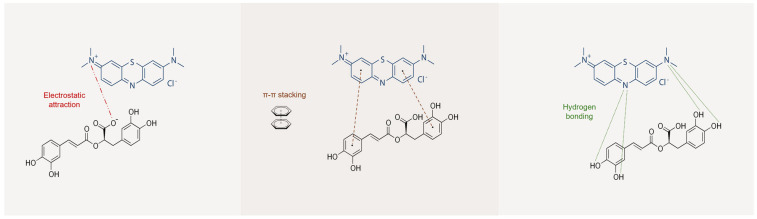
Schematic illustration on the interactions between methylene blue and rosmarinic acid (the most abundant compound in plant extracts) under acidic and basic conditions.

Another interaction that warrants consideration is π-π stacking, which is independent of pH. Phenol groups of rosmarinic acid and MB exhibit sp^2^ hybridization at the carbon atoms in the benzene ring and at the oxygen atom of the –OH group. This hybridization promotes the interaction of the π-electrons from the phenolic structures with the π-electrons of the dye molecules [58]. The π–π stacking interactions are indirectly supported by ATR-FTIR results (Figure S3), which show a slight blue shift and decreased intensity of the C=C stretching band of the extract (~1500 cm^−1^) in the composite beads. A schematic illustration of the major interactions between rosmarinic acid and MB under acidic and basic conditions is provided in Figure 5. In the present study, phenolics were encapsulated in SA, an anionic natural copolymer composed of β-D-mannuronic and α-L-guluronic acids. According to Allangawi et al. [59], the deprotonation of the carboxylic acid groups of SA at high pH has been identified as a key factor contributing to the effectiveness of MB adsorption. Similar to phenolics, the interaction between these deprotonated carboxylic groups and MB occurs primarily through strong electrostatic interactions [59].

It is important to note that, although the ATR-FTIR analysis provided valuable insights into the functional group interactions involved in MB adsorption, the compositional complexity and overlapping bands limit its resolution in such systems. To overcome these limitations, future studies will incorporate complementary techniques, such as solid-state 2D-NMR and advanced surface characterization, to provide a more robust and detailed understanding of the underlying interaction mechanisms.

#### 2.3.2. Adsorption Conditions Optimization Using BBD-RSM

To optimize MB removal efficiency, fifteen experiments generated by the BBD–RSM approach (Table S3) were conducted to assess the influence of three independent variables [pH (A; values from 4 to 10), MB concentration (B; ranged from 1000 to 3000 mg/L), and adsorbent mass (C; between 1.9 mg or 1 bead and 5.7 mg or 3 beads)] on the quantity of MB adsorbed (q_ads_, mg/g). These experiments were performed using the “richest” extract-loaded hydrogels, which exhibit the highest total phenolic content (Table 2), for each plant species and micropropagation stage, resulting in a total of four models. The coefficients were analyzed using the method of analysis of variance (ANOVA). As described in Table S4, the *p*-value and F-value were calculated to evaluate the significance and effectiveness of the proposed response model. All independent variables (A, B, C) demonstrated a significant influence on the four models, with *p*-values lower than 0.05. Among these, MB concentration (B) was the most dominant factor, exhibiting the highest F-values (29.06–663.23), followed by adsorbent mass (C) and pH (A).

**Figure 6:**
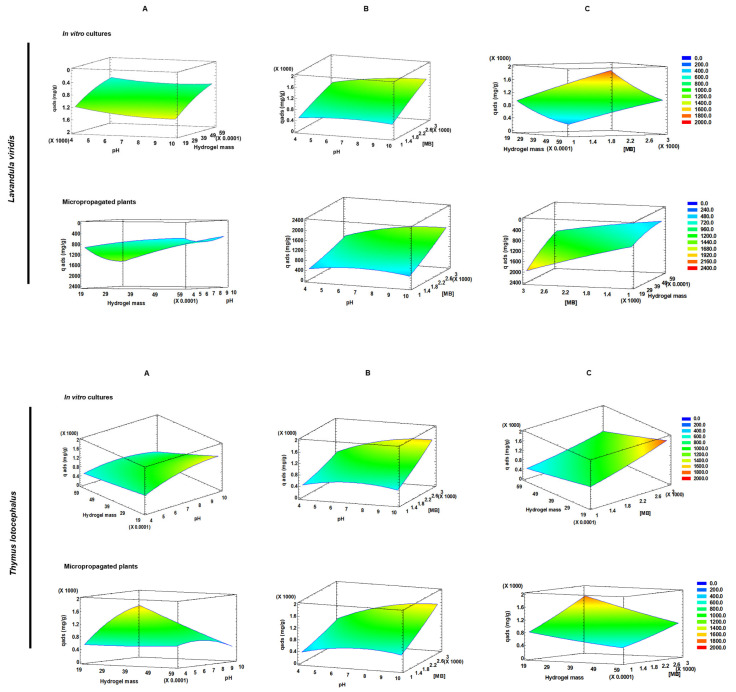
Estimated response surface for the quantity of methylene blue adsorbed (mg/g), alginate beads incorporating extracts from in vitro cultures and micropropagated plants of *Lavandula viridis* and *Thymus lotocephalus* subjected to two distinct UV-B treatments (UV-B 1 and UV-B 4). This was analyzed using a two–factorial Box–Behnken design with Response Surface Methodology: (**A**). hydrogel mass [1.9 mg (1 bead)–3.8 mg (2 beads)–5.7 mg (3 beads)] vs. pH (4–7–10), (**B**). pH vs. MB concentration (1000–2000–3000 mg/L), and (**C**). hydrogel mass vs. MB concentration.

The effect of interaction variables on the removal efficiency of MB is depicted in the 3D surface graphs of Figure 6. The four extract-loaded hydrogels tested exhibited similar behavior, with the maximum MB dye removal efficiency achieved at pH 10, an MB concentration of 3000 mg/L, and the use of one hydrogel bead. Specifically, the conditions were as follows: *L. viridis* IC, pH 9.69, [MB] 3000 mg/L, hydrogel mass 1.9 mg; *L. viridis* MP, pH 10, [MB] 3000 mg/L, hydrogel mass 2.1 mg; *T. lotocephalus* IC, pH 10, [MB] 3000 mg/L, hydrogel mass 2.0 mg; and *T. lotocephalus* MP, pH 10, [MB] 2947.8 mg/L, hydrogel mass 1.9 mg. According to the Pareto charts (Figure S4), both [MB] and pH positively influenced all four models, while hydrogel mass did not. Furthermore, the interaction between hydrogel mass and either [MB] or pH negatively impacted MB removal.

A decrease in adsorbent concentration in a solution at a given adsorbate concentration increases the adsorbate/adsorbent ratio, thereby enhancing adsorption [60]. This assertion is validated in the present study, where the models employed were based on the quantity (milligrams) of MB adsorbed per gram of adsorbent (mg/g). Notably, the optimal conditions were achieved with a single bead, underscoring the efficacy of the experimental design. At lower concentrations of the adsorbent, a greater number of available adsorption sites per MB molecule is observed, thereby enabling enhanced individual adsorbent saturation. Conversely, as the concentration of adsorbent increases, the q_ads_ decreases, likely due to a dilution effect, where aggregation or reduced surface area exposure diminishes the number of adsorption sites per unit of hydrogel [61]. These results are in agreement with previous studies on MB adsorption. However, studies reporting adsorption as a percentage of removal have shown that increasing adsorbent dosage enhances MB adsorption [60,62]. This discrepancy arises because the percentage of removal solely considers the initial and equilibrium (final) concentrations of the solution. In this case, a lower adsorbent dosage results in the saturation of binding sites, which reduces adsorption. This phenomenon also applies to the initial MB concentration when expressed as removal percentage. As the initial dye concentration increases, adsorption sites on the adsorbent surface become saturated, leading to a lower removal percentage [4]. However, in the present study, the q_ads_ increases with the increase in the initial MB concentration due to mass transfer driving force; a high MB concentration in the bulk solution and a low concentration at the adsorbent surface generate a concentration gradient. The greater the gradient, the higher the adsorption as MB molecules migrate toward the adsorbent surface to balance the concentration levels [3].

Regarding pH, although it exerted the least influence on MB adsorption, the results are consistent with the pH_PZC_ measurements, confirming that the most alkaline pH is optimal [1,3,4].

Although adsorption kinetics and isotherm modeling are essential tools for elucidating adsorption mechanisms, this study focused specifically on the optimization of adsorption conditions using BBD–RSM. Therefore, at this stage, the experimental design did not incorporate broader concentration ranges or time-resolved data typically required for such analyses.

#### 2.3.3. Impact of Green Extracts from UV-B Stressed Lamiaceae Species on MB Adsorption Under Optimal Conditions

**Figure 7:**
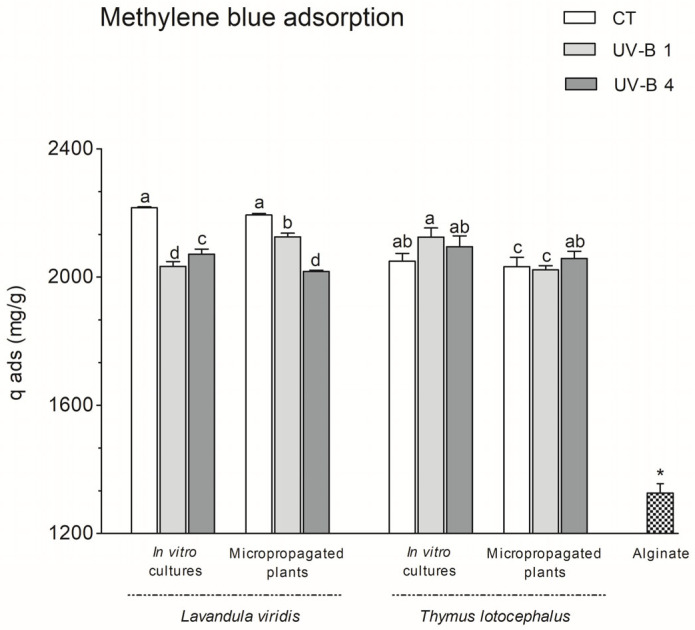
Quantity of methylene blue adsorbed (mg/g) by alginate beads incorporating extracts (extract-loaded hydrogel) from in vitro cultures and micropropagated plants of *Lavandula viridis* and *Thymus lotocephalus* subjected to two distinct UV-B treatments (UV-B 1 and UV-B 4), under the optimal conditions verified by Response Surface Methodology. For each species, the results were analyzed using one-way analysis of variance (ANOVA), and the graph bars followed by different letters (a–d) are significantly different at *p* < 0.05 (Duncan’s New Multiple Range Test). (*) To compare isolated alginate with each extract-loaded hydrogel, a Dunnett *t*-test was performed at *p* < 0.05.

Following the establishment of the optimal conditions, the MB adsorption capacity of hydrogels loaded with green extracts from IC and MP of *L. viridis* and *T. lotocephalus* treated with UV-B radiation was evaluated and compared with the control IC and MP. The extracts were incorporated into SA beads and the q_ads_ of MB (mg/g) were analyzed using the optimal removal conditions established by BBD–RSM: an adsorbent mass of 1.9 mg (1 bead), an MB concentration of 3000 mg/L, and a pH of 10. SA is an anionic natural copolymer that is known to be an efficient adsorbent for MB [63,64]. In this study, all extract-loaded hydrogels demonstrated significantly higher q_ads_ and % removal (%R) in comparison to the hydrogel composed solely of SA (without plant extracts) (1327 ± 28.92 mg/g, %R = 41%), enhancing the MB adsorption by 52.09% to 67.01% (Figure 7). The hydrogels that exhibited the highest q_ads_ of MB were those containing extracts from the control IC and MP of *L. viridis* (IC: 2216 ± 3.25 mg/g, %R = 68% and MP: 2194 ± 4.43 mg/g, %R = 67%).

The impact of UV-B treatments on the MB adsorption capacity of the extract-loaded hydrogels was also investigated, revealing a 3.08% to 8.21% decrease in capacity, especially in *L. viridis*. This decrease may be partly attributed to the UV-B-induced decline in the levels of specific phenolic compounds, such as salvianolic acid I (isomer IV), rabdosiin hexoside, salviaflaside (isomer II), and yunnaneic acid F. Although these are minor constituents, they may play a key role in the adsorption process due to their structural characteristics and reactivity. Moreover, plant extracts are inherently complex mixtures in which synergistic and antagonistic interactions among compounds can modulate overall adsorption performance. The effects of UV-B treatment on IC and MP were examined, with the former showing no effect on the hydrogel but a slight improvement in MP adsorption. Only two studies in the literature have explored the potential of the *Lavandula* and *Thymus* genera for MB adsorption. In the study by Li et al. [21], *Lavandin* hydrochar was produced through hydrothermal carbonization and treated with NaOH to increase its surface oxygen content, aiming to enhance MB adsorption. The highest MB adsorption (306 mg/g) was observed when the *Lavandin* hydrochar was subjected to 4% NaOH. In another study, Youcefi et al. [20] investigated the removal of MB from aqueous solutions by *Thymus numidicus* leaf residues, achieving maximum adsorption (41 mg/g) at neutral pH and ambient temperature. Although direct comparisons with existing literature may be complex, the phenolic extract-loaded hydrogel developed in this study exhibits a remarkable maximum adsorption capacity of 2216 mg/g, surpassing several other SA-based adsorbents documented in the literature (Table 3). In the present study, Pearson’s correlation (Figure 2) demonstrated that the compounds rosmarinic acid, theaflavic acid, yunnaneic acid F, methylrosmarinic acid, salvianolic B, and salvianolic A, present in the extracts of both species, contribute most significantly to the increased removal of MB. Additionally, the flavone luteolin-7-*O*-glucuronide was identified as a significant compound in the extracts of *T. lotocephalus*. The presence of hydroxyl and carboxyl groups in the structures of these compounds enhances their hydrophilicity, thereby facilitating interactions with MB, a highly water-soluble dye [13].

Beyond the previously discussed interactions of phenolics-MB and SA-MB (Section 2.3.1), the NADES used for extracting these biocompounds may also play a significant role in dye adsorption. Arcon and Franco [71] demonstrated that fatty acid-based hydrophobic deep eutectic solvent mixtures effectively removed various toxic industrial dyes, including MB. More recently, J. Wang et al. [72] compared the MB removal efficacy of polyaniline@Fe_3_O_4_ magnetic microspheres with and without different DES in their composition. The study demonstrated that the incorporation of DES not only enhanced the adsorbent surface area but also provided numerous active sites for effective dye removal and facilitated rapid MB mass transfer. Notably, the NADES mixture that demonstrated the most promising capacity to remove MB was choline chloride: lactic acid, the same NADES used in the present study to extract the phenolic compounds.

Overall, the chemical structures of all components involved in the synthesis of the hydrogel, namely phenolics, NADES, and SA, exhibited favorable properties to enhance MB adsorption. The stability of adsorption complexes involving phenolic compounds is significantly enhanced by their antioxidant properties, which help to maintain the integrity of adsorptive sites and prevent degradation [73]. Moreover, this antioxidant activity could also ensure the hydrogel’s long-term stability. In this regard, the antioxidant activity of the phenolic extracts examined was previously demonstrated [17]. Although the antioxidant properties of the incorporated extracts suggest potential long-term stability, this study did not experimentally evaluate the hydrogels’ reusability or durability, which should be explored in future investigations.

## 3. Materials and Methods

### 3.1. Chemicals and Reagents

Lactic acid was provided by Acros Organics (Geel, Germany). Hydrogen peroxide (H_2_O_2_), trichloroacetic acid (TCA), thiobarbituric acid (TBA), calcium chloride dihydrate, HPLC-MS-grade water, HPLC-MS-grade acetonitrile, luteolin, epigallocatechin gallate, protocatechuic acid and formic acid were obtained from Sigma–Aldrich (Steinheim, Germany). Rosmarinic acid and quercetin were acquired from Extrasynthese (Genay, France), and ρ-coumaric, caffeic acid, and catechin were supplied by AASC Ltd. (Southampton, UK). 6-Benzyladenine (BA) and calcium chloride were purchased from Fluka (Buchs, Switzerland). Sucrose, ethanol, sulfuric acid, choline chloride, sodium alginate, methylene blue, phenol and acetone were provided by VWR (Radnor, Pennsylvania) and plant agar was obtained from Duchefa Biochemie (Haarlem, The Netherlands). Potassium di-hydrogen phosphate and di-potassium hydrogen phosphate were supplied by Panreac (Barcelona, Spain).

### 3.2. In Vitro Culture, Micropropagation and UV-B Treatment

IC of *T. lotocephalus* and *L. viridis* were obtained from the Plant Biotechnology Laboratory, Faculty of Sciences and Technology, University of Algarve (Faro, Portugal). The cultures were multiplied on hormone-free Murashige and Skoog (MS) medium [74] and MS supplemented with 0.2 mg/L 6-benzyladenine (BA), respectively, according to the protocols of Dias et al. [75] and Coelho et al. [76]. Both media contained 2% (*w*/*v*) sucrose and 0.7% (*w*/*v*) agar and were autoclaved at 121 °C for 20 min. Cultures were incubated at 25 ± 2 °C under a 16 h light cycle for 7 weeks. MP were produced from these IC according to Nogueira and Romano [77] and were acclimatized and grown under ex vitro conditions for 16 months before being subjected to the experimental treatments.

Control cultures and plants of both species were grown in a growth chamber (750 E, Aralab, Lisbon, Portugal) fitted with four cool white lamps (Osram L 18W/840), under a 16 h light/8 h dark photoperiod at 25 ± 2 °C and 50–60% relative humidity. For UV-B treatments, the cultures and plants were maintained in the same chamber and irradiated with four UV-B lamps (Philips TL 20W/12 RS SLV/25, 290–315 nm) placed 30 cm above the plant material. The radiation was measured using an Analytik Jena AG UVX Radiometer (Jena, Germany). Two distinct exposure periods were tested: UV-B 1 (i.e., 4 h for 1 day, 4 J/cm^2^) or UV-B 4 (i.e., 4 h for 4 days, 16 J/cm^2^). These periods were selected based on preliminary tests with various UV-B exposure periods (30 min, 4 h, 8 h, and 16 h per day, over 1 or 4 consecutive days). Leaves were visually examined for signs of damage. No visible damage was observed after 30 min/day of exposure, regardless of treatment duration. Mild damage began to appear at 4 h/day, while more pronounced and progressive leaf damage was observed from 8 h/day onwards, increasing with both the daily exposure time and the total treatment duration. Magnified areas highlight visible symptoms such as darkening and necrotic lesions, particularly along the leaf margins and central veins (Figure S1A). Based on these findings, the two least-damaging exposure durations (30 min and 4 h per day) were selected for subsequent analyses aimed at determining their effect on phenolic compound accumulation (Figure S1B). Among these, the 4 h treatments induced the highest phenolic content without causing significant physiological stress. Consequently, the selected exposure regimens (UV-B 1 and UV-B 4) are considered sub-lethal doses capable of effectively enhancing secondary metabolism without inducing substantial tissue damage.

IC were maintained in wide-neck borosilicate glass Pyrex Erlenmeyer flasks. This type of glass has an optical cutoff wavelength below 300 nm, effectively blocking harmful UV-C radiation while transmitting more than 80% at the UV-B wavelengths emitted by the lamps used in this study (Philips TL 20W/12 RS). It also allows high transmittance of longer UV-B wavelengths, as well as UV-A and visible light, thereby ensuring biologically relevant UV-B exposure of the plant material [78]. After each UV-B treatment, a portion of the plant material (in vitro shoots and aerial parts of micropropagated plants) was frozen in liquid nitrogen and stored at −80 °C until further use, while the remaining material was oven-dried at 40 °C.

### 3.3. Total Chlorophyll and Carotenoids Contents

The contents of chlorophyll and carotenoids were evaluated following the protocol described by Lichtenthaler (1987) [79]. In brief, photosynthetic pigments were extracted by macerating 25 mg of plant material in 4 mL of pure acetone. The resulting extract was then centrifuged, and the absorbance of the supernatant was measured at three distinct wavelengths (i.e., 470, 644.8, and 661.6 nm) using a T70+ UV/Vis Spectrophotometer (PG Instruments Ltd., Leicestershire, UK). The results were expressed as milligrams per gram of fresh weight.

### 3.4. Oxidative Stress Signs

The levels of hydrogen peroxide (H_2_O_2_) and malondialdehyde (MDA) were evaluated according to Mansinhos et al. [17]. In brief, fresh plant material (ca. 100 mg) was mixed with 1 mL of 0.1% (*w*/*v*) TCA, centrifuged, and the supernatant was used to assess both H_2_O_2_ and MDA contents. In the H_2_O_2_ assay, 0.2 mL of supernatant was mixed with 0.2 mL of potassium phosphate buffer (10 mM). The absorbance was read at 390 nm 30 min later using a microplate reader (SynergyTM HTX MultiMode Microplate Reader, BioTek Instruments, Inc., Winooski, VT, USA) and the results were presented as micromoles of H_2_O_2_ equivalents per gram of fresh weight (µmol_H2O2_/g_FW_). To estimate the level of lipid peroxidation (MDA content), the supernatant (0.5 mL) was mixed with an equal volume of either positive [TCA (5%, *w*/*v*) + TBA (20%, *w*/*v*)] or negative control solutions [TCA (20%, *w*/*v*)] and the obtained mixtures were incubated at 95 °C for 30 min. To stop the reaction, the mixtures were placed on ice and the absorbance measured (600, 532, and 440 nm). The final results were expressed as nanomoles of MDA equivalents per gram of fresh weight (nmol_MDA_/g_FW_).

### 3.5. Soluble Sugars Content

The protocol described by Abid et al. [80] for the phenol-sulfuric acid method was followed to evaluate the concentration of soluble sugars. Plant material weighing 25 mg was extracted with 2 mL of 80% (*v*/*v*) ethanol in a water bath set at 80 °C for 30 min. The supernatant (100 µL) was diluted in distilled water (1:1) and combined with phenol (9%, *w*/*v*) (200 µL) and pure sulfuric acid (1000 µL). After a 30 min incubation period at room temperature, the absorbance was measured at 490 nm using the microplate reader. The results were expressed as milligrams of glucose equivalents per gram of fresh weight (mg_GLU_/g_FW_).

### 3.6. Phenolics Extraction and HPLC–HRMS-Based Phenolic Metabolite Profiling

To perform a green extraction, the dried plant material described in Section 3.2 was ground to a size of less than 2 mm using a shiver. The ground material (250 mg) was mixed with 10 mL of a previously prepared Natural Deep Eutectic Solvent (NADES) composed of choline chloride: lactic acid at a 1:2 ratio, with 30% (*w*/*w*) water, and placed in an Ultrasound-Assisted Extraction apparatus (Elmasonic S 100 (H), Elma Hans Schmidbauer GmbH & Co. KG, Singen, Germany) for 30 min at 50 °C [81]. The mixture was passed through a Whatman filter No. 1 (Whatman Int. Ltd., Maidstone, UK) and the extracts were stored at −20 °C until use.

To evaluate the phenolic profile of the plant extracts, an HPLC-HRMS analysis was conducted according to Mansinhos et al. [18]. A Dionex Ultimate 3000 HPLC system (Thermo Scientific, San Jose, CA, USA) equipped with an autosampler and an HPLC pump operating at 10 °C was used for the analysis. Samples were separated using a 100 Kinetex C18 column (150 × 4.6 mm i.d., 5 µm) (Phenomenex, Macclesfield, UK) at 40 °C and a flow rate of 1 mL/min. The solvent system consisted of two mobile phases: Milli-Q double-distilled water (solvent A) and acetonitrile (solvent B), both containing 0.1% formic acid. The gradient was as follows: 0 min—90% A; 10 min—74% A; 22 min—35% A; 30 min—5% A; 40 min—5% A; 40.1 min—90% A; and 45 min—90% A. An Exactive Orbitrap mass spectrometer (Thermo Fisher Scientific, San José, CA, USA) with a heated electrospray ionization probe (HESI) operated at a column flow rate of 0.2 mL/min. To investigate negative ions, an auto 100–1000 *m*/*z* MS/MS scan mode was employed. Full-scan mass spectra were acquired using two microscans at a resolution of 50,000 and an automatic gain control (AGC) target of one million charges. In-source collision-induced dissociation scans (25 eV) were performed using a spray voltage of 4000 V, a heater temperature of 150 °C, a capillary temperature of 320 °C, 25 units of sheath gas flow, and 5 units of auxiliary gas. Data acquisition and processing were carried out using Xcalibur software (version 3.0; Thermo Fisher Scientific, San Jose, CA, USA). The Exactive Orbitrap was externally calibrated weekly using ready-to-use calibration mixtures of Pierce LQT ESI Positive Ion Calibration Solution and Pierce ESI Negative Ion Calibration Solution (Thermo Fisher Scientific, San Jose, CA, USA). A quality control (QC) sample was utilized to assess and validate the accuracy of the analytical process. This QC sample consisted of identical aliquots from a representative pool of the plant extracts. The QC sample was frequently injected during the run to monitor drifts and calculate the variance of metabolite characteristics (below 20%). Standards were used to identify the compounds based on retention time and exact mass. If the standards were unavailable, the substance was initially identified by comparing the theoretically precise molecular ion mass with the defined accurate mass of the molecular ion. Subsequently, it was cross-referenced with several metabolite databases, including ChemSpider, Metlin, Phenol Explorer, and PubChem. The identification of the phenolic profile was performed using the MSI MS levels previously defined by Sumner et al. [82]. Table S1 provides the list of the compounds’ chemical formulas, theoretical precise masses, delta ppm values, retention times (RT), and MSI identification levels. Table S2 summarizes the assumptions made to quantify the phenolic compounds, including linear range, intercept, slope, R^2^, limits of detection (LOD), and limits of quantification (LOQ). The LOD and LOQ were found to be 2.06–153.40 µg/L and 6.24–464.85 µg/L, respectively. The exact data are presented in milligrams per gram of dry weight (mg/g_DW_) or micrograms per gram of dry weight (µg/g_DW_) in Table 1.

### 3.7. Plant Extract-Loaded Hydrogels for Adsorption of Methylene Blue

#### 3.7.1. Preparation of Hydrogels

The sodium alginate (SA)/plant extract beads (hydrogel) were prepared according to the procedure described by Chen et al. [83], with slight modifications. Firstly, SA was dissolved in distilled water at a concentration of 4% (*w*/*v*) under mechanical stirring at 60 °C until complete dissolution. The hydrogels obtained were used as controls. Hydrogels were prepared by mixing 2% (*w*/*v*) of the plant extracts with 2% (*w*/*v*) of SA and vortexing until a homogeneous solution was obtained. Both the control and the hydrogels were formed by precipitating hydrogel droplets into a 4% (*w*/*v*) CaCl_2_ solution, using a syringe. To minimize variability in bead size and shape associated with syringe-based dripping methods, we standardized key operational parameters, including the use of a fixed needle gauge, a consistent dropping height, and a controlled flow rate. The beads were left at room temperature for 24 h to ensure complete crosslinking. To remove any unreacted excess CaCl_2_, the hydrogels were rinsed five times with distilled water and stored in a refrigerator until further use.

#### 3.7.2. Point of Zero Charge (pH_PZC_)

The pH_PZC_ was determined according to Djebri et al. [84]. The initial pH (pH_i_) of NaCl solutions (0.01 M, 1.5 mL) was adjusted to a range of 2–10 using HCl (0.1 M) or NaOH (0.1 M). Then, three beads (ca. 6 mg) of each sample were added to solutions with different pH values. The dispersions were stirred for 24 h at room temperature, and the final pH (pH_f_) of the solutions was measured. Finally, the pH_PZC_ was determined by plotting ∆pH (pH_f_ − pH_i_) versus pH_i_.

#### 3.7.3. MB Adsorption Assay

The MB adsorption behavior of the hydrogel was investigated, focusing on the influence of various variables: initial adsorbate (MB) concentration, solution pH, and adsorbent (phenolic-loaded hydrogel) dosage. The evaluation of MB adsorption quantity (q_ads_, mg/g) was conducted according to Musa et al. [62]. In each flask, different doses of the adsorbent hydrogel [ranging from 1.9 (1 bead) to 5.7 mg (3 beads)] were added to 2 mL of the adsorbate solution (MB) with concentrations varying from 1000 to 3000 mg/L and pH values between 4 and 10. The solution was continuously agitated in an orbital shaker incubator at room temperature, and the absorbance was measured after 24 h at 664 nm. The initial and final (equilibrium) MB concentrations were obtained from the MB calibration curve (y = 0.1712x + 0.0282) with R^2^ = 0.9979. The adsorbed quantities at equilibrium, q_e_ (mg/g), were determined according to the following equation:

(1) $$q_{e}=\frac{\left(C_{0}-C_{e}\right)\cdot V}{m}$$

where *C*_0_ and *C_e_* are the initial and equilibrium adsorbate concentrations (mg/L), respectively; *V* is the volume (L) of the solution; and *m* is the mass (g) of the dry adsorbent.

#### 3.7.4. Box–Behnken Optimization for MB Removal

The Box–Behnken design (BBD), a multivariate statistical method based on the principles of response surface methodology (RSM), was employed to optimize the MB adsorption process. The second-order polynomial equation relating to the model’s response is presented in Equation (2), where Y represents the response variable (i.e., MB adsorption quantity in mg/g), *β*_0_ is the intercept, X_i_ corresponds to the variables evaluated during the experimental design, *β_i_* represents the linear coefficients, *β_ii_* denotes the quadratic coefficients, and *β_ij_* refers to the cross-product coefficients.

(2) $$Y=\beta_{0}+\sum \beta_{i}X_{i}+\sum \beta_{ii}X_{i}^{2}+\sum \beta_{ij}X_{i}X_{j}$$

According to this method, three levels (low–medium–high) have been assigned to each variable. In this particular case, three variables were selected within the following ranges: pH (4–7–10), MB concentration (1000–2000–3000 mg/L), and hydrogel mass [1.9 mg (1 bead)–3.8 mg (2 beads)–5.7 mg (3 beads)]. The reaction time was fixed at 24 h. These conditions and ranges were chosen based on the prior experience of the research group. In total, 15 experiments were performed (Table S3), each in duplicate and in a random order. These experiments were repeated for one representative extract sample from IC and MP of *L. viridis* and *T. lotocephalus*, resulting in a total of four samples. The representative samples were selected based on their highest total phenolic content, as quantified by HPLC-HRMS (Table 1). The BBD-RSM experimental design was developed, and the results were subsequently analyzed using Statgraphics Centurion software version 18 (Warrenton, VA, USA) at a 95% confidence level.

#### 3.7.5. Characterization by ATR-FTIR

The vibrational modes of the main compounds presents in the individual components (SA, MB, and extract), binary systems (SA + MB, SA + extract), and the ternary bead composite (SA + MB + extract) were assessed by attenuated total reflectance–Fourier transform infrared (ATR-FTIR) spectroscopy using a Nicolet Summit X spectrometer (Thermo Fisher Scientific, Waltham, MA, USA) equipped with a diamond ATR crystal. Spectra were collected with 24 scans at a resolution of 4 cm^−1^ in the range of 4000–400 cm^−1^, using the OMNIC Paradigm software version 2.7, with data points acquired every 300 nm. Prior to analysis, the systems were dried at 50 °C, ground, and sieved to obtain fine powders suitable for spectral acquisition.

### 3.8. Statistical Analysis

Data are presented as mean ± standard error (SE) of three biological replicates (*n* = 3). Statistical analyses were performed using one-way analysis of variance (ANOVA), followed by Duncan’s multiple range test (*p* < 0.05). Multivariate analyses, including Venn diagrams, hierarchical clustering, polar heatmaps, and heatmaps, were performed using OriginPro 2022 (OriginLab Corporation, Northampton, MA, USA).

## 4. Conclusions

This report presents the findings of the first investigation into the impact of UV-B radiation on *L. viridis* and *T. lotocephalus*. The study revealed that UV-B radiation induced oxidative stress in both species, leading to an increased production of phenolic compounds, with rosmarinic acid being the most predominant. Furthermore, plant extracts obtained using a NADES were incorporated into an SA-based hydrogel and successfully used for the removal of MB from water. A BBD-RSM was employed to examine the effects of three variables—pH, MB concentration, and adsorbent dosage—on the MB adsorption process and to optimize the adsorption conditions. Although this is a preliminary study on the effectiveness of plant extracts in MB adsorption, the results are promising and suggest that the development of this adsorbent holds great potential for providing a sustainable solution for contaminated water treatment, as it is composed entirely of biodegradable natural materials and is easy to produce without high energy costs. Furthermore, the phenolics present in the hydrogel are extracted from plants derived from in vitro culture technique, an approach that prevents any negative impact on the natural populations of the species. Additionally, the use of underexplored aromatic species from the Mediterranean region could contribute to the sustainable valorization of natural resources. As the field progresses, future studies will investigate adsorption kinetics and isotherms to further enhance the understanding of the interactions and adsorption mechanisms between the adsorbate and adsorbent. In addition, the performance of these beads over multiple adsorption–desorption cycles will be evaluated to confirm their suitability for practical and sustainable wastewater treatment applications. Comprehensive morphological and chemical characterization of hydrogels will also be conducted to further assess and boost their potential.

## Figures and Tables

**Table 1:** Effect of two distinct UV-B treatments (UV-B 1 and UV-B 4) on hydrogen peroxide (H_2_O_2_), malondialdehyde (MDA) and soluble sugar contents in in vitro cultures and micropropagated plants of *Lavandula viridis* and *Thymus lotocephalus*.

	*Lavandula viridis*						*Thymus lotocephalus*					
	In Vitro			Micropropagated			In Vitro			Micropropagated		
	CT	UV-B 1	UV-B 4	CT	UV-B 1	UV-B 4	CT	UV-B 1	UV-B 4	CT	UV-B 1	UV-B 4
H_2_O_2_ (µmol/g_FW_)	2.13 ± 0.01 b	2.20 ± 0.13 b	1.12 ± 0.08 d	1.49 ± 0.07 c	2.57 ± 0.03 a	1.57 ± 0.06 c	0.33 ± 0.02 d	0.31 ± 0.00 d	0.52 ± 0.06 d	2.34 ± 0.16 c	4.18 ± 0.08 b	4.70 ± 0.04 a
MDA (nmol/g_FW_)	10.35 ± 0.82 c	10.02 ± 1.20 c	5.06 ± 0.57 d	13.45 ± 1.09 b	31.64 ± 0.32 a	15.07 ± 0.04 b	4.66 ± 0.31 c	8.43 ± 1.44 b	4.82 ± 0.53 c	8.92 ± 0.15 b	8.87 ± 0.86 b	17.45 ± 1.04 a
Soluble sugars (mg/g_FW_)	20.72 ± 0.33 bc	42.16 ± 3.47 a	37.89 ± 1.52 a	18.62 ± 1.43 c	24.91 ± 1.52 b	43.73 ± 1.00 a	14.02 ± 0.65 d	25.12 ± 2.10 b	24.06 ± 1.72 bc	16.56 ± 0.63 cd	24.06 ± 2.39 bc	56.40 ± 4.76 a

For each species, the results were analyzed using one-way analysis of variance (ANOVA). Graph bars followed by different letters (a–d) indicate significant differences at *p* < 0.05 (Duncan’s New Multiple Range Test).

**Table 2:** Quantitative (µg/g_DW_ or mg/g_DW_ *; mean ± SE) analysis by HPLC-HRMS of the phenolic profile from in vitro cultures and micropropagated plant extracts of *Lavandula viridis* and *Thymus lotocephalus* exposed to two distinct UV-B treatments (UV-B 1 and UV-B 4).

Compound	*Lavandula viridis*						*Thymus lotocephalus*					
	In Vitro			Micropropagated			In Vitro			Micropropagated		
	CT	UV-B 1	UV-B 4	CT	UV-B 1	UV-B 4	CT	UV-B 1	UV-B 4	CT	UV-B 1	UV-B 4
**Phenolic acids**												
Salvianolic acid A isomer I	790.5 ± 5.3 c	195.6 ± 0.4 f	918.2 ± 0.6 a	451.4 ± 2.3 d	873.5 ± 21.2 b	395.8 ± 1.2 e	648.5 ± 4.0 c	532.5 ± 10.7 d	652.6 ± 9.9 c	1096.3 ± 20.5 a	537.7 ± 2.5 d	991.0 ± 0.1 b
Salvianolic acid A isomer II	168.3 ± 6.5 a	50.8 ± 2.1 c	184.3 ± 0.8 a	125.6 ± 7.5 b	69.2 ± 0.5 c	108.8 ± 10.6 b	231.1 ± 4.7 ab	139.7 ± 10.6 d	240.0 ± 8.6 a	211.8 ± 2.5 b	163.4 ± 3.6 c	243.7 ± 0.8 a
Salvianolic acid A isomer III	<LOQ	<LOQ	<LOQ	21.5 ± 0.1	66.9 ± 1.2	35.3 ± 2.8	107.7 ± 3.1 d	171.3 ± 4.0 c	161.6 ± 4.0 c	465.4 ± 13.1 b	460.5 ± 2.9 b	605.6 ± 14.7 a
Salvianolic acid B isomer I	18.7 ± 0.2	<LOQ	20.4 ± 0.5	n.d.	n.d.	n.d.	115.7 ± 1.2 a	57.2 ± 0.4 c	86.2 ± 0.3 b	<LOD	29.8 ± 0.7 d	<LOD
Salvianolic acid B isomer II	n.d.	n.d.	n.d.	115.0 ± 0.6	117.4 ± 3.8	n.d.	480.6 ± 2.4 c	349.4 ± 2.0 d	161.8 ± 4.2 e	778.8 ± 10.8 a	366.7 ± 4.0 d	661.8 ± 3.5 b
Salvianolic acid B isomer III	183.1 ± 2.1 cd	185.5 ± 1.1 c	168.1 ± 3.9 e	237.2 ± 3.6 b	419.1 ± 1.1 a	176.5 ± 0.5 d	72.8 ± 3.0 d	119.7 ± 0.2 b	90.1 ± 4.7 c	118.8 ± 0.8 b	125.4 ± 10.2 ab	141.1 ± 2.2 a
Salvianolic acid B isomer IV	* 12.6 ± 0.0 b	* 4.2 ± 0.0 e	* 13.9 ± 0.3 a	* 6.8 ± 0.1 c	* 4.6 ± 0.1 e	* 5.7 ± 0.0 d	191.3 ± 0.6 a	133.0 ± 1.5 b	118.4 ± 4.1 c	185.6 ± 0.8 a	124.9 ± 5.2 bc	134.9 ± 2.6 b
Salvianolic acid B isomer V	n.d.	n.d.	n.d.	n.d.	n.d.	n.d.	518.6 ± 52.4 a	313.0 ± 0.7 b	303.1 ± 32.1 b	284.2 ± 9.1 b	189.5 ± 4.3 c	236.6 ± 7.3 bc
Salvianolic acid B isomer VI	n.d.	16.2 ± 2.0 c	n.d.	47.5 ± 1.2 a	44.4 ± 0.5 a	35.6 ± 0.7 b	206.4 ± 0.7 d	185.9 ± 1.7 d	66.9 ± 3.2 e	858.6 ± 1.5 b	370.6 ± 5.6 c	1086.1 ± 14.8 a
Salvianolic acid F	247.9 ± 7.3 c	280.7 ± 3.7 b	329.5 ± 12.3 a	44.2 ± 3.0 e	38.1 ± 0.4 e	69.1 ± 2.8 d	84.1 ± 0.6 c	82.9 ± 1.8 c	27.0 ± 0.4 e	66.2 ± 2.4 d	120.5 ± 0.2 a	95.4 ± 2.9 b
Salvianolic acid I isomer I	28.9 ± 0.3 a	21.2 ± 1.2 b	32.6 ± 1.8 a	n.d.	n.d.	n.d.	n.d.	n.d.	n.d.	n.d.	n.d.	n.d.
Salvianolic acid I isomer II	51.0 ± 0.1 b	23.0 ± 0.3 c	82.8 ± 0.2 a	<LOQ	<LOQ	<LOQ	53.8 ± 0.3 a	42.3 ± 0.8 c	47.7 ± 0.8 b	n.d.	<LOQ	n.d.
Salvianolic acid I isomer III	529.2 ± 3.5 d	258.1 ± 0.8 f	717.8 ± 0.5 c	867.2 ± 12.8 a	420.1 ± 31.8 e	768.1 ± 3.6 b	<LOQ	n.d.	n.d.	n.d.	n.d.	n.d.
Salvianolic acid I isomer IV	320.8 ± 7.4 b	144.4 ± 1.5 d	404.7 ± 1.9 a	198.2 ± 2.4 c	97.3 ± 3.1 f	122.6 ± 4.2 e	186.8 ± 0.9 e	231.4 ± 2.1 d	274.4 ± 3.6 b	329.3 ± 4.0 a	199.8 ± 1.2 e	256.6 ± 7.2 c
Salvianolic acid I isomer V	176.3 ± 4.0 a	27.8 ± 1.6 e	145.8 ± 6.9 b	49.8 ± 1.4 d	82.2 ± 0.2 c	52.2 ± 1.3 d	n.d.	n.d.	n.d.	n.d.	n.d.	n.d.
Salvianolic acid I isomer VI	340.3 ± 0.5 b	124.5 ± 2.8 f	396.6 ± 10.7 a	304.1 ± 6.1 c	163.9 ± 1.7 e	258.4 ± 7.8 d	465.7 ± 2.9 c	293.5 ± 4.3 e	454.1 ± 0.7 c	499.0 ± 1.7 b	380.4 ± 8.9 d	563.4 ± 12.1 a
* **Total salvianolic acids**	15.5 ± 0.0 b	5.6 ± 0.0 f	17.3 ± 0.3 a	9.3 ± 0.1 c	7.0 ± 0.0 e	7.8 ± 0.1 d	3.4 ± 0.1 b	2.7 ± 0.0 d	2.7 ± 0.0 d	4.9 ± 0.1 a	3.1 ± 0.0 c	5.0 ± 0.0 a
Caffeic acid	123.4 ±1.4 d	190.5 ± 0.7 b	136.5 ± 1.0 d	159.0 ± 1.0 c	661.1 ± 9.5 a	191.9 ± 1.3 b	92.1 ± 0.0 d	96.6 ± 3.2 cd	222.2 ± 0.8 a	86.6 ± 3.3 d	121.8 ± 0.9 b	103.9 ± 5.5 c
* Dimethyl lithospermate B	2.7 ± 0.3 a	2.8 ± 0.0 a	2.7 ± 0.4 a	3.0 ± 0.0 a	1.7 ± 0.0 b	3.1 ± 0.0 a	2.7 ± 0.4 ab	2.6 ± 0.2 ab	2.0 ± 0.0 b	2.4 ± 0.0 ab	2.8 ± 0.0 a	2.5 ± 0.0 ab
Fertaric acid	73.3 ± 1.2 e	176.8 ± 0.5 d	64.6 ± 1.9 e	464.6 ± 8.8 b	361.0 ± 4.1 c	501.7 ± 3.1 a	4.6 ± 0.0 a	2.6 ± 0.0 b	n.d.	n.d.	1.8 ± 0.0 c	n.d.
Methylrosmarinic acid isomer I	381.9 ± 7.1 b	233.5 ± 2.0 d	421.2 ± 8.0 a	313.8 ± 5.6 c	412.2 ± 7.9 a	165.4 ± 0.8 e	67.4 ± 1.7 c	81.8 ± 1.1 b	89.9 ± 1.5 a	31.4 ± 2.9 d	26.7 ± 0.4 de	24.3 ± 0.9 e
Methylrosmarinic acid isomer II	851.6 ± 54.2 a	962.1 ± 66.4 a	843.2 ± 48.3 a	522.0 ± 30.3 b	479.1 ± 19.5 bc	358.5 ± 23.9 c	464.3 ± 26.1 cd	577.6 ± 38.4 bc	486.0 ± 33.1 cd	639.7 ± 34.2 ab	384.6 ± 21.2 d	713.4 ± 42.3 a
Monomethyl lithospermate	35.7 ± 1.5 b	18.2 ± 0.8 d	45.1 ± 1.9 a	24.1 ± 0.8 c	25.5 ± 0.8 c	26.2 ± 0.8 c	n.d.	n.d.	n.d.	<LOQ	n.d.	<LOD
Rabdosiin hexoside	43.3 ± 1.5 a	29.4 ± 1.0 c	34.9 ± 0.4 b	<LOQ	<LOQ	<LOQ	n.d.	n.d.	n.d.	n.d.	n.d.	n.d.
* Rosmarinic acid	43.1 ± 0.3 b	50.1 ± 0.2 a	43.1 ± 0.1 b	20.4 ± 0.1 d	23.1 ± 0.2 c	15.7 ± 0.1 e	23.0 ± 0.2 d	32.3 ± 0.0 a	26.5 ± 0.2 b	24.0 ± 0.0 c	17.4 ± 0.1 e	26.6 ± 0.1 b
Salviaflaside isomer I	68.6 ± 0.3 c	98.0 ± 0.7 a	75.4 ± 0.4 b	30.7 ± 0.2 f	33.4 ± 0.4 e	35.6 ± 0.2 d	39.5 ± 0.5 b	49.3 ± 0.4 a	31.6 ± 0.2 c	27.4 ± 0.3 d	<LOQ	30.9 ± 0.2 c
Salviaflaside isomer II	168.4 ± 2.3 c	278.8 ± 1.0 a	196.8 ± 2.5 b	115.9 ± 1.2 d	66.1 ± 3.8 e	69.8 ± 1.9 e	67.1 ± 8.7 abc	77.8 ± 13.1 abc	58.6 ± 7.8 bc	103.5 ± 24.9 ab	54.7 ± 2.9 c	110.2 ± 0.8 a
Yunnaneic acid D	<LOQ	<LOQ	<LOQ	<LOQ	<LOQ	<LOQ	n.d.	n.d.	n.d.	n.d.	n.d.	n.d.
Yunnaneic acid F	43.7 ± 0.3 e	52.0 ± 0.2 d	43.7 ± 1.1 e	170.1 ± 0.5 a	146.7 ± 2.2 b	139.3 ± 0.1 c	80.5 ± 1.9 d	118.3 ± 2.8 c	85.9 ± 0.5 d	183.4 ± 1.1 b	177.7 ± 4.1 b	202.6 ± 3.8 a
* **Total phenolic acids**	63.1 ± 0.0 b	60.5 ± 0.3 c	65.0 ± 0.2 a	34.5 ± 0.1 d	34.0 ± 0.3 d	28.0 ± 0.1 e	29.9 ± 0.1 d	38.6 ± 0.3 a	32.2 ± 0.1 c	32.4 ± 0.0 c	24.0 ± 0.0 e	35.3 ± 0.0 b
**Flavonoids**												
Dihydromorelloflavone	102.6 ± 1.6 ab	92.4 ± 1.5 b	111.5 ± 4.1 a	99.1 ± 1.3 ab	93.1 ± 2.3 b	109.6 ± 9.4 a	116.8 ± 16.5 a	112.3 ± 10.7 a	95.9 ± 0.3 a	124.3 ± 12.6 a	100.9 ± 3.8 a	99.8 ± 0.1 a
Epigallocatechin gallate	121.8 ± 1.4 b	125.3 ± 0.3 ab	133.4 ± 4.7 a	125.8 ± 2.3 ab	97.0 ± 2.6 c	131.6 ± 0.8 a	134.0 ± 2.9 a	129.7 ± 1.6 ab	110.1 ± 0.9 c	114.6 ± 3.0 bc	125.7 ± 3.4 abc	117.4 ± 10.4 abc
Luteolin	3.6 ± 0.1	3.6 ± 0.0	n.d.	n.d.	n.d.	n.d.	6.7 ± 0.2 c	6.2 ± 0.1 c	6.9 ± 0.0 c	6.2 ± 0.1 c	9.4 ± 0.2 a	7.6 ± 0.3 b
Luteolin-7-*O*-glucuronide	30.1 ± 0.7 b	25.8 ± 0.1 bc	40.5 ± 3.7 a	13.2 ± 0.5 d	24.2 ± 0.6 bc	20.9 ± 1.4 c	240.3 ± 2.6 ab	298.6 ± 28.9 a	249.9 ± 2.0 ab	198.1 ± 21.4 bc	172.7 ± 14.7 c	246.5 ± 21.9 ab
Theaflavic acid	1129.4 ± 9.2 b	1189.5 ± 0.7 a	1113.0 ± 0.1 b	695.9 ± 4.0 d	777.3 ± 8.6 c	547.1 ± 7.5 e	733.3 ± 0.8 d	927.7 ± 11.3 a	845.8 ± 13.6 b	749.7 ± 4.9 d	594.0 ± 3.9 e	795.8 ± 8.9 c
* **Total flavonoids**	1.4 ± 0.0 b	1.4 ± 0.0 a	1.4 ± 0.0 b	0.9 ± 0.0 d	1.0 ± 0.0 c	0.8 ± 0.0 e	1.2 ± 0.0 cd	1.5 ± 0.0 a	1.3 ± 0.0 b	1.2 ± 0.0 d	1.0 ± 0.0 e	1.3 ± 0.0 bc
**Galloyl esters**												
* Methyl-*O*-galloyl-D-glucopyranoside	14.9 ± 0.4 a	12.5 ± 0.4 b	14.9 ± 0.5 a	15.0 ± 0.1 a	8.9 ± 0.2 c	13.3 ± 0.6 b	12.2 ± 0.1 a	10.5 ± 0.1 b	8.0 ± 0.0 c	11.9 ± 0.3 a	10.6 ± 0.4 b	11.8 ± 0.1 a
**Coumarin derivative**												
Herniarin	73.2 ± 0.0 c	45.6 ± 1.0 e	81.5 ± 1.0 b	58.7 ± 1.6 d	93.8 ± 0.5 a	24.3 ± 2.0 f	5.7 ± 0.9 b	8.1 ± 1.6 b	13.7 ± 0.9 a	1.6 ± 0.0 c	1.0 ± 0.2 c	0.7 ± 0.1 c
**Hydroxybenzaldehyde**												
Protocatechuic aldehyde	221.1 ± 4.4 d	237.9 ± 8.0 d	218.6 ± 4.2 d	281.0 ± 11.5 c	651.2 ± 1.2 a	311.0 ± 3.8 b	174.4 ± 6.9 e	205.3 ± 0.2 c	236.7 ± 5.0 b	182.7 ± 4.5 de	318.2 ± 2.7 a	194.4 ± 2.2 cd
* **Total phenolic compounds**	79.7 ± 0.4 b	74.7 ± 0.1 c	81.6 ± 0.2 a	50.8 ± 0.2 d	44.6 ± 0.4 e	42.5 ± 0.5 f	43.5 ± 0.2 d	50.8 ± 0.4 a	41.7 ± 0.1 e	45.6 ± 0.3 c	35.9 ± 0.5 f	48.5 ± 0.2 b

Notes: CT—control; n.d.—not detected; LOD—limit of detection; LOQ—limit of quantification. The results were analyzed using one-way analysis of variance (ANOVA) followed by Duncan’s New Multiple Range Test. Different letters (a to f) in each phenolic compound and for each plant species mean significant differences (*p* < 0.05) among treatments.

**Table 3:** Comparison of the MB removal performance with other SA-based adsorbents.

Adsorbents	Adsorption Capacity (mg/g)	Refs.
Polyaniline-SA	1060.78	[65]
Carboxymethyl chitosan-SA	1010	[66]
Modified biochar-SA	1373.49	[67]
Clay-SDS-SA	1468.5	[68]
SA-g-p(AA-co-St)/organo-illite/smectite clay	1841	[69]
Graphene-SA	2300	[70]
Phenolic extract-SA	2216	This work

## Data Availability

The original contributions presented in this study are included in the article. Further inquiries can be directed to the corresponding authors.

## Supplementary Materials

The following supporting information can be downloaded at: https://www.mdpi.com/article/10.3390/plants15142210/s1, Figure S1. (A): Preliminary assessment of UV-B radiation effects on *Lavandula viridis* micropropagated plants. Plants were exposed to UV-B radiation for different daily durations (30 min, 4 h, 8 h, and 16 h per day) over 1 or 4 consecutive days. The control plant (CT), shown on the left, was not exposed to UV-B radiation.; (B): Preliminary assay of total phenolic content, evaluated using the Folin–Ciocalteu method, in extracts from *Lavandula viridis* and *Thymus lotocephalus* in vitro cultures (IC) and micropropagated plants (MP).; Figure S2. Point of Zero Charge (pH_pzc_) of polyphenol-loaded hydrogels derived from in vitro cultures (IC) and micropropagated plants (MP) of *Lavandula viridis* (L) and *Thymus lotocephalus* (T).; Table S1. HPLC-HRMS data of identified phenolic compounds in *Lavandula viridis* and *Thymus lotocephalus* extracts.; Table S2. Summary of HPLC-HRMS criteria for quantification of phenolic compounds in *Lavandula viridis* and *Thymus lotocephalus* extracts.; Table S3. Box–Behnken designs used to optimize the methylene blue (MB) removal capacity (adsorption quantity, mg/g) by alginate beads with incorporated extracts from in vitro cultures and micropropagated plants of *Lavandula viridis* and *Thymus lotocephalus*.; Table S4. Analysis of variance (ANOVA) of the quadratic model adjusted to the quantity of methylene blue (MB) adsorbed (mg/g) by alginate beads with incorporated extracts from in vitro cultures and micropropagated plants of *Lavandula viridis* and *Thymus lotocephalus*.; Figure S3. (A) ATR-FTIR spectra of (A) the individual components sodium alginate (SA), methylene blue (MB) and plant extract; (B) binary systems (SA + MB, SA + extract); and (C) the ternary composite (SA + MB + extract).; Figure S4. Pareto chart results from the quantity of methylene blue (MB) adsorbed (mg/g) during Box–Behnken design with response surface methodology. A: pH; B: methylene blue (MB) concentration; C: hydrogel mass. D. The vertical line represents 95% confidence. The gray bars reflect the positive influence (+) while the black bars show the negative effect (−).

## Abbreviations

The following abbreviations are used in this manuscript:

BBD	Box–Behnken Design
BBD-RSM	Box–Behnken Design–Response Surface Methodology
H_2_O_2_	Hydrogen Peroxide
HPLC-HRMS	High-Performance Liquid Chromatography–High Resolution Mass Spectrometry
IC	In vitro cultures
MB	Methylene Blue
MDA	Malondialdehyde
MP	Micropropagated plants
NADES	Natural Deep Eutectic Solvents
pH_PZC_	Point of Zero Charge
ROS	Reactive Oxygen Species
RSM	Response Surface Methodology
SA	Sodium alginate
UV-B	Ultraviolet-B Radiation
UV-B 1	Single 4 h UV-B Exposure
UV-B 4	Repeated UV-B Exposure for Four Consecutive Days
