# Supplementary material for: UV-B Radiation Triggers Phenolic Accumulation and Oxidative Stress Response in Lamiaceae Species: From Plant Defense to Green Dye Remediation
Source: Plants (Basel). 2026 Jul 20;15(14):2210. doi: 10.3390/plants15142210 (PMC13416476; doi:10.3390/plants15142210)

UV-B Radiation Triggers Phenolic Accumulation and Oxidative Stress Response in Lamiaceae Species: From Plant Defense to Green Dye Remediation

Inês Mansinhos [1], Sandra Gonçalves [1,*], João Brás [1], Raquel Rodríguez-Solana [1,2], María José Aliaño Gonzalez [1,3], Bruno Medronho [1,4], Gema Pereira-Caro [5,6], José Manuel Moreno-Rojas [5,6] and Anabela Romano [1]

[1] MED—Mediterranean Institute for Agriculture, Environment and Development & CHANGE—Global Change and Sustainability Institute, Faculdade de Ciências e Tecnologia, Universidade do Algarve, Campus de Gambelas, 8005-139 Faro, Portugal; ifmansinhos@ualg.pt (I.M.); jtbras@hotmail.com (J.B.); raquel.rodriguez.solana@juntadeandalucia.es (R.R.-S.); mariajose.aliano@gm.uca.es (M.J.A.G.); bfmedronho@ualg.pt (B.M.); aromano@ualg.pt (A.R.)

[2] Department of Agroindustry and Food Quality, Andalusian Institute of Agricultural and Fisheries Research and Training (IFAPA), Rancho de la Merced Center, Carretera Cañada de la Loba (CA-3102) Km 3.1., SN, 11471 Jerez de la Frontera, Cádiz, Spain

[3] Analytical Chemistry Department, University of Cádiz, 11510 Puerto Real, Cádiz, Spain

[4] Surface and Colloid Engineering, FSCN Research Center, Mid Sweden University, SE-851 70 Sundsvall, Sweden

[5] Department of Agroindustry and Food Quality, Andalusian Institute of Agricultural and Fisheries Research and Training (IFAPA), Alameda del Obispo Center, Avenida Menendez-Pidal, SN, 14004 Córdoba, Córdoba, Spain; mariag.pereira@juntadeandalucia.es (G.P.-C.); josem.moreno.rojas@juntadeandalucia.es (J.M.M.-R.)

[6] Foods for Health Group, Instituto Maimónides de Investigación Biomédica de Córdoba (IMIBIC), Avenida Menendez-Pidal, SN, 14004 Córdoba, Córdoba, Spain

* Correspondence: smgoncalves@ualg.pt

Supplementary information

Figure S1. A) Preliminary assessment of UV-B radiation effects on *Lavandula viridis* micropropagated plants. Plants were exposed to UV-B radiation for different daily durations (30 minutes, 4 hours, 8 hours, and 16 hours per day) over 1 or 4 consecutive days. The control plant (CT), shown on the left, was not exposed to UV-B radiation.

A

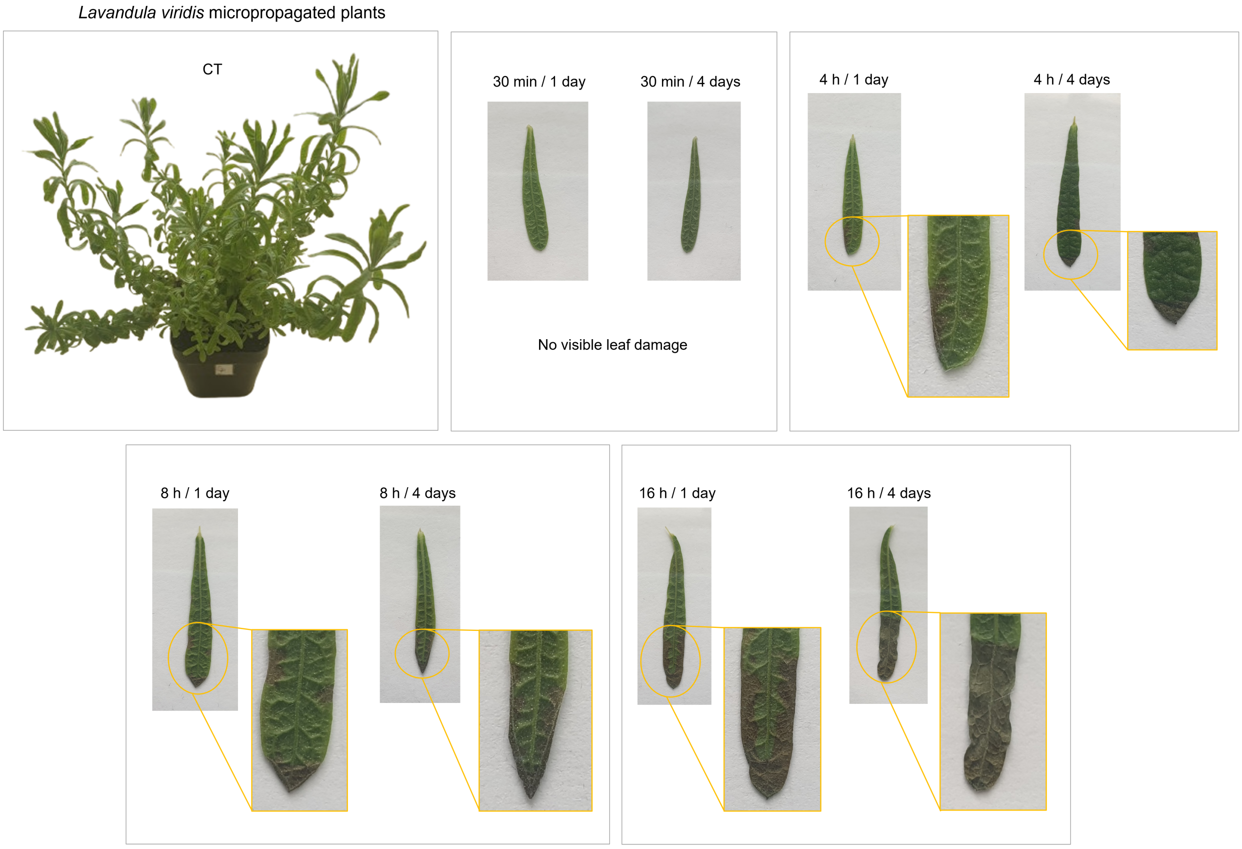

Figure S1. B) Preliminary assay of total phenolic content, evaluated using the Folin–Ciocalteu method, in extracts from *Lavandula viridis* and *Thymus lotocephalus in vitro* cultures (IC) and micropropagated plants (MP). Results are expressed as milligrams of gallic acid equivalents (GAE) per gram of dry weight (mg_{GAE}/g_{DW}).

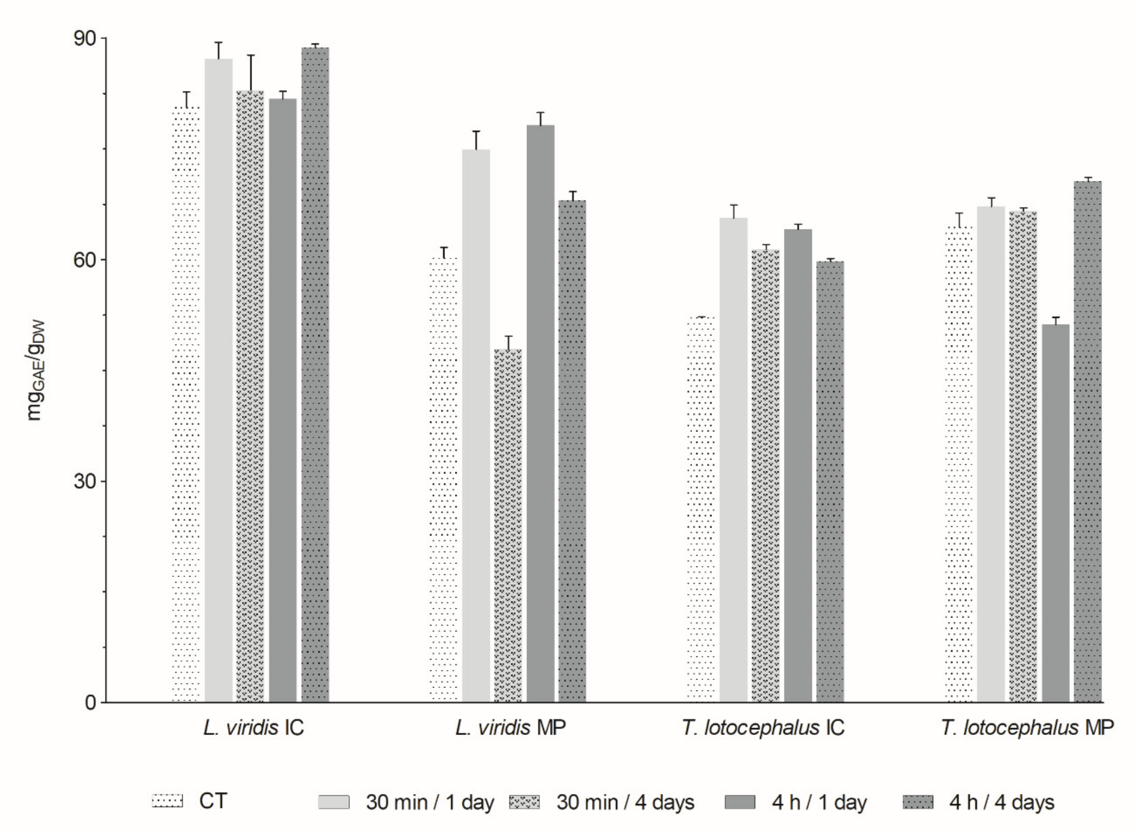

Figure S2. Point of Zero Charge (pH_{pzc}) of polyphenol-loaded hydrogels derived from *in vitro* cultures (IC) and micropropagated plants (MP) of *Lavandula viridis* (L) and *Thymus lotocephalus* (T).

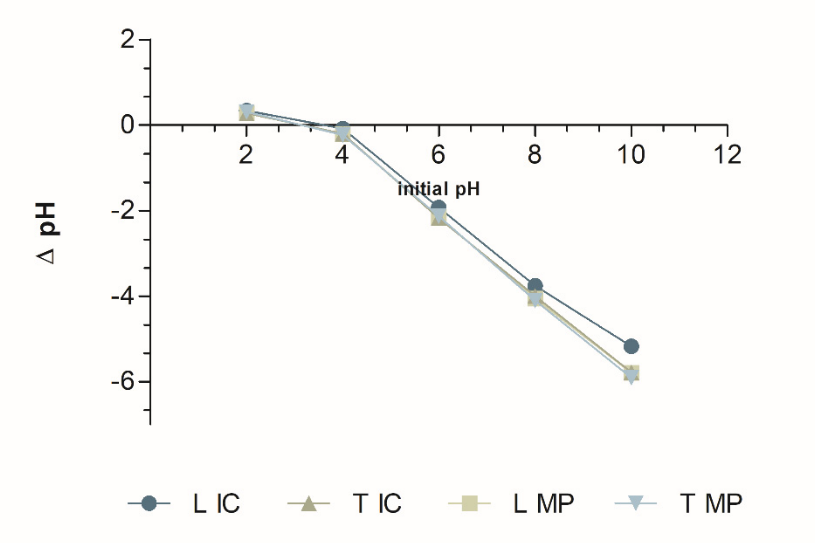

Table S1. HPLC-HRMS data of identified phenolic compounds in *Lavandula viridis* and *Thymus lotocephalus* extracts.

Compound identity	Chemical formula	Theoretical exact mass $[M-H]^-$ *(m/z)*	Delta ppm (error)	RT (min)	MSI MI level*
Phenolic acids					
Caffeic acid	$C_9H_8O_4$	179.0338	-1.481	4.55	1
Dimethyl lithospermate B [1]	$C_{38}H_{34}O_{16}$	745.1763	-0.055	4.57	2
Fertaric acid [2]	$C_{14}H_{14}O_9$	325.0554	-1.472	4.25	2
Methylrosmarinic acid isomer I [1]	$C_{19}H_{18}O_8$	373.0918	0.258	12.04	2
Methylrosmarinic acid isomer II [1]	$C_{19}H_{18}O_8$	373.0918	0.097	13.53	2
Monomethyl lithospermate [1]	$C_{28}H_{24}O_{12}$	551.1184	-0.513	12.58	2
Rabdosiin hexoside [1]	$C_{42}H_{40}O_{21}$	879.1978	-1.757	9.42	2
Rosmarinic acid	$C_{18}H_{16}O_8$	359.0761	0.88	9.57	1
Salviaflaside isomer I [1 c]	$C_{24}H_{26}O_{13}$	521.1290	-0.705	7.07	2
Salviaflaside isomer II [1 c]	$C_{24}H_{26}O_{13}$	521.1290	-1.165	8.81	2
Salvianolic acid A isomer I [1]	$C_{26}H_{22}O_{10}$	493.1129	-0.453	10.03	2
Salvianolic acid A isomer II [1]	$C_{26}H_{22}O_{10}$	493.1129	-0.635	10.96	2
Salvianolic acid A isomer III [1]	$C_{26}H_{22}O_{10}$	493.1129	-0.879	12.93	2
Salvianolic acid B isomer I [1 b]	$C_{36}H_{30}O_{16}$	717.1450	-0.671	5.99	2
Salvianolic acid B isomer II [1 b]	$C_{36}H_{30}O_{16}$	717.1450	-0.587	8.81	2
Salvianolic acid B isomer III [1 b]	$C_{36}H_{30}O_{16}$	717.1450	-0.308	9.59	2
Salvianolic acid B isomer IV [1 b]	$C_{36}H_{30}O_{16}$	717.1450	-0.239	11.03	2
Salvianolic acid B isomer V [1 b]	$C_{36}H_{30}O_{16}$	717.1450	-0.838	12.92	2
Salvianolic acid B isomer VI [1 b]	$C_{36}H_{30}O_{16}$	717.1450	-1.173	14.17	2
Salvianolic acid F [1]	$C_{17}H_{14}O_6$	313.0707	0.528	15.04	2
Salvianolic acid I isomer I [1 a]	$C_{27}H_{22}O_{12}$	537.1027	-1.755	3.2	2

Salvianolic acid I isomer II [1 a]	$C_{27}H_{22}O_{12}$	537.1027	-0.507	3.82/4.01	2
Salvianolic acid I isomer III [1 a]	$C_{27}H_{22}O_{12}$	537.1027	-0.377	6.07	2
Salvianolic acid I isomer IV [1 a]	$C_{27}H_{22}O_{12}$	537.1027	-0.731	6.85	2
Salvianolic acid I isomer V [1 a]	$C_{27}H_{22}O_{12}$	537.1027	0.517	10.02	2
Salvianolic acid I isomer VI [1 a]	$C_{27}H_{22}O_{12}$	537.1027	-0.731	11.03	2
Yunnaneic acid D [1]	$C_{27}H_{24}O_{12}$	539.1184	-0.672	7	2
Yunnaneic acid F [1]	$C_{29}H_{26}O_{14}$	597.1239	-0.891	7.1	2
Flavonoids					
Dihydromorelloflavone [4]	$C_{30}H_{22}O_{11}$	557.1078	0.237	4.92	2
Epigallocatechin gallate	$C_{22}H_{18}O_{11}$	457.0765	-5.493	4.87	1
Luteolin	$C_{15}H_{10}O_{6}$	285.0394	3.528	15.35	1
Luteolin-7-*O*-glucuronide [5]	$C_{21}H_{18}O_{12}$	461.0714	-0.959	8.57	2
Theaflavic acid [6]	$C_{21}H_{16}O_{10}$	427.0660	-6,915	9.57	2
Galloyl esters					
Methyl-*O*-galloyl-D-glucopyranoside [3]	$C_{14}H_{18}O_{10}$	345.0816	-7.399	2.37	2
Couramin derivatives					
Herniarin [7]	$C_{10}H_{8}O_{3}$	175.0390	-1.375	12.03	2
Hydroxybenzaldehydes					
Protocatechuic aldehyde [8]	$C_{7}H_{6}O_{3}$	137.0239	0.07	3.96	2

*Metabolite standards initiative metabolite identification (MSI MI) levels. Reference standards were available for all compounds identified at MSI MI level 1. Results expressed as equivalents of rosmarinic acid (1), caffeic acid (2), gallic acid (3), quercetin (4), luteolin (5), catechin (6), *p*-coumaric acid (7), protocatechuic acid (8).

Notes: (a) Salvianolic acid I (synonym: Melitric acid A) possible isomers: Salvianolic acid H, Lithospermic acid A, Clinopodic acid C, Clinopodic acid E, Yunnaneic acid C. (b) Salvianolic acid B (synonym: Lithospermic acid B) possible isomers: Salvianolic acid L, Salvianolic acid E, Salvianolic acid Y, Rabdosiin, Yunnaneic acid G. (c) Salviaflaside (synonym: Rosmarinic acid 3-glucoside) possible isomers: Monomethyl lithospermate, methyl melitrate A.

Table S2. Summary of HPLC-HRMS criterion for quantification of phenolic compounds in *Lavandula viridis* and *Thymus lotocephalus* extracts.

Compound	Linear range (mg/L)	Intercept	Slope	R^2	LOD (μg/L)	LOQ (μg/L)
Caffeic acid	0.03-1.60	13434.730	3689287.210	0.9995	2.22	6.74
Catechin	0.20-12.50	55997.686	1785701.714	0.9998	19.08	57.82
p-Coumaric acid	0.03-1.60	44526.027	1703452.384	0.9988	3.03	9.17
Epigallocatechin gallate	0.10-3.10	135286.063	1045329.093	0.9990	9.63	29.18
Gallic acid	3.10-70.00	1432483.723	1910268.617	0.9996	3.94	11.93
Luteolin	0.03-0.20	37177.957	4402412.870	0.9993	9.68	29.32
Protocatechuic acid	0.05-12.50	292967.588	1831200.109	0.9979	2.06	6.24
Quercetin	0.05-3.10	131148.385	2623736.111	0.9997	11.45	34.69
Rosmarinic acid	0.025-100.00	799442.221	1926874.899	0.9994	153.40	464.85

Table S3. Box–Behnken designs used to optimize the methylene blue (MB) removal capacity (adsorption quantity, mg/g) by alginate beads with incorporated extracts from *in vitro* cultures and micropropagated plants of *Lavandula viridis* and *Thymus lotocephalus*.

Sample 1: *Lavandula viridis, in vitro* cultures, UV-B 4

Experiment	pH	[MB] (mg/L)	Hydrogel mass (g)	q ads (mg/g) observed	q ads (mg/g) Adjusted	Error (%)
1	7	2000	0.0038	1011.00	982.333	2.836
2	4	1000	0.0038	448.00	474.250	5.859
3	10	3000	0.0038	1475.00	1448.750	1.780
4	10	1000	0.0038	476.00	486.000	2.101
5	4	3000	0.0038	1094.00	1084.000	0.914
6	7	1000	0.0057	333.00	337.375	1.314
7	7	3000	0.0019	1821.00	1816.630	0.240
8	10	2000	0.0057	695.00	680.625	2.068
9	10	2000	0.0019	1437.00	1467.630	2.132
10	4	2000	0.0019	1171.00	1185.380	1.228
11	4	2000	0.0057	617.00	586.375	4.964
12	7	2000	0.0038	947.00	982.333	3.731
13	7	2000	0.0038	989.00	982.333	0.674
14	7	3000	0.0057	995.00	1035.630	4.083
15	7	1000	0.0019	983.00	942.375	4.133

Sample 2: *Lavandula viridis,* micropropagated plants, CT

Experiment	pH	[MB] (mg/L)	Hydrogel mass (g)	q ads (mg/g) observed	q ads (mg/g) Adjusted	Error (%)
1	7	2000	0.0038	975.00	980.333	0.547
2	4	1000	0.0038	514.00	443.125	13.789
3	10	3000	0.0038	1504.00	1574.870	4.712
4	10	1000	0.0038	485.00	443.125	8.634
5	4	3000	0.0038	917.00	958.875	4.567
6	7	1000	0.0057	328.00	382.750	16.692
7	7	3000	0.0019	2119.00	1964.250	7.303
8	10	2000	0.0057	686.00	573.125	16.454
9	10	2000	0.0019	1427.00	1510.870	5.877
10	4	2000	0.0019	810.00	922.875	13.935
11	4	2000	0.0057	629.00	545.125	13.335
12	7	2000	0.0038	973.00	980.333	0.754
13	7	2000	0.0038	993.00	980.333	1.276
14	7	3000	0.0057	1003.00	1045.000	4.187
15	7	1000	0.0019	921.00	879.000	4.560

Sample 3: *Thymus lotocephalus, in vitro* cultures, UV-B 1

Experiment	pH	[MB] (mg/L)	Hydrogel mass (g)	q ads (mg/g) observed	q ads (mg/g) Adjusted	Error (%)
1	7	2000	0.0038	988.00	990.000	0.202
2	4	1000	0.0038	506.00	431.250	14.773
3	10	3000	0.0038	1505.00	1579.750	4.967
4	10	1000	0.0038	478.00	497.000	3.975
5	4	3000	0.0038	932.00	913.000	2.039
6	7	1000	0.0057	327.00	358.125	9.518
7	7	3000	0.0019	1954.00	1822.880	6.710
8	10	2000	0.0057	675.00	524.875	22.241
9	10	2000	0.0019	1423.00	1479.380	3.962
10	4	2000	0.0019	591.00	741.125	25.402
11	4	2000	0.0057	587.00	530.625	9.604
12	7	2000	0.0038	995.00	990.000	0.503
13	7	2000	0.0038	987.00	990.000	0.304
14	7	3000	0.0057	981.00	1056.380	7.684
15	7	1000	0.0019	932.00	856.625	8.087

Sample 4: *Thymus lotocephalus,* micropropagated plants, UV-B 4

Experiment	pH	[MB] (mg/L)	Hydrogel mass (g)	q ads (mg/g) observed	q ads (mg/g) Adjusted	Error (%)
1	7	2000	0.0038	963.00	969.000	0.623
2	4	1000	0.0038	506.00	363.125	28.236
3	10	3000	0.0038	1415.00	1557.870	10.097
4	10	1000	0.0038	431.00	510.125	18.358
5	4	3000	0.0038	910.00	830.875	8.695
6	7	1000	0.0057	329.00	367.250	11.626
7	7	3000	0.0019	1891.00	1752.750	7.311
8	10	2000	0.0057	682.00	664.625	2.548
9	10	2000	0.0019	1593.00	1588.370	0.291
10	4	2000	0.0019	338.00	355.375	5.141
11	4	2000	0.0057	619.00	623.625	0.747
12	7	2000	0.0038	959.00	969.000	1.043
13	7	2000	0.0038	985.00	969.000	1.624
14	7	3000	0.0057	977.00	1051.500	7.625
15	7	1000	0.0019	896.00	821.500	8.315

Table S4. Analysis of variance (ANOVA) of the quadratic model adjusted to the quantity of methylene blue (MB) adsorbed (mg/g) by alginate beads with incorporated extracts from *in vitro* cultures and micropropagated plants of *Lavandula viridis* and *Thymus lotocephalus*.

Sample 1: *Lavandula viridis, in vitro* cultures, UV-B 4

Variable	Sum of Squares	F–Value	*p*–Value
A: pH	70876.10	38.02	**0.00**
B: [MB]	1236380.00	**663.23**	**0.00**
C: Hydrogel mass	960498.00	515.24	**0.00**
AA	24250.20	13.01	**0.02**
AB	31152.30	16.71	**0.01**
AC	8836.00	4.74	0.08
BB	2903.39	1.56	0.27
BC	7744.00	4.15	0.10
CC	22873.90	12.27	**0.02**
Total error	9320.92		

Sample 2: *Lavandula viridis,* micropropagated plants, CT

Variable	Sum of Squares	F–Value	*p*–Value
A: pH	189728	9.05	**0.03**
B: [MB]	1357130	**64.77**	**0.00**
C: Hydrogel mass	865270	41.29	**0.00**
AA	100574	4.80	0.08
AB	94864	4.53	0.09
AC	78400	3.74	0.11
BB	5821.85	0.28	0.62
BC	68382.2	3.26	0.13
CC	19519.4	0.93	0.38
Total error	104771		

Sample 3: *Thymus lotocephalus, in vitro* cultures, UV-B 1

Variable	Sum of Squares	F–Value	*p*–Value
A: pH	268278	12.29	**0.017**
B: [MB]	1223830	**56.08**	**0.001**
C: Hydrogel mass	678612	31.10	**0.003**
AA	122472	5.61	0.064
AB	90300.2	4.14	0.098
AC	138384	6.34	0.053

BB	8286.98	0.38	0.565
BC	33856	1.55	0.268
CC	456.981	0.02	0.891
Total error	109117		

Sample 4: *Thymus lotocephalus*, micropropagated plants, UV-B 4

Variable	Sum of Squares	F–Value	*p*–Value
A: pH	381938	9.66	**0.027**
B: [MB]	1148370	29.06	**0.003**
C: Hydrogel mass	557040	14.09	**0.013**
AA	125517	3.18	0.135
AB	84100	2.13	0.204
AC	355216	8.99	**0.030**
BB	3519.75	0.09	0.777
BC	30102.2	0.76	0.423
CC	2017.44	0.05	0.830
Total error	197613		

Figure S3. (A) ATR-FTIR spectra of (A) the individual components sodium alginate (SA), methylene blue (MB) and plant extract; (B) binary systems (SA+MB, SA+extract); and (C) the ternary composite (SA+MB+extract).

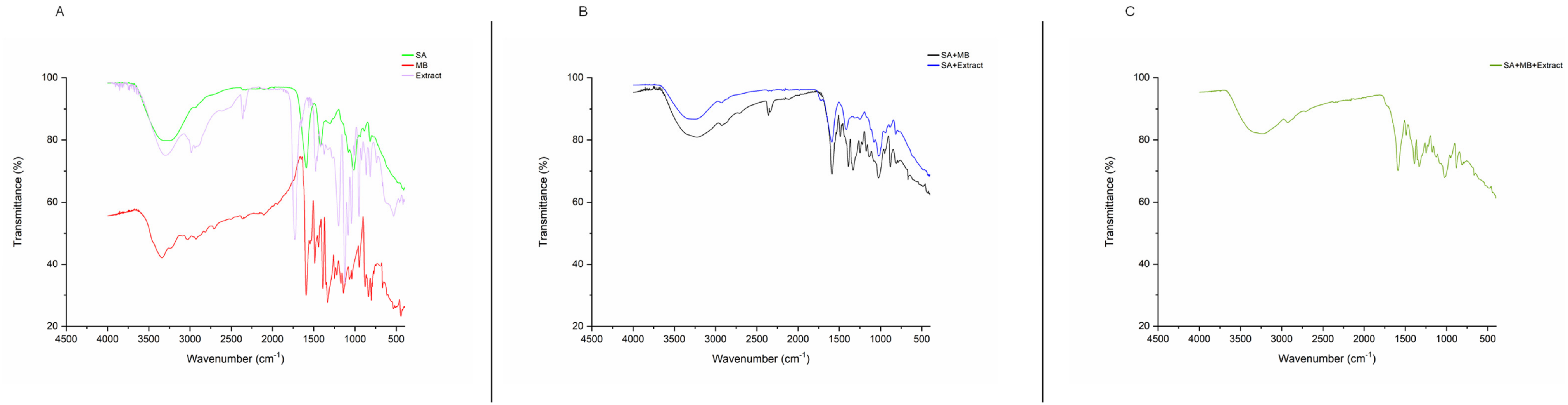

Figure S4. Pareto chart results from the quantity of methylene blue (MB) adsorbed (mg/g) during Box-Behnken design with response surface methodology. **A:** pH; **B**: methylene blue (MB) concentration; **C**: hydrogel mass. **D**. The vertical line represents 95% confidence. The gray bars reflect the positive influence (+) while the black bars show the negative effect (−).

Sample 1: *Lavandula viridis, in vitro* cultures, UV-B 4

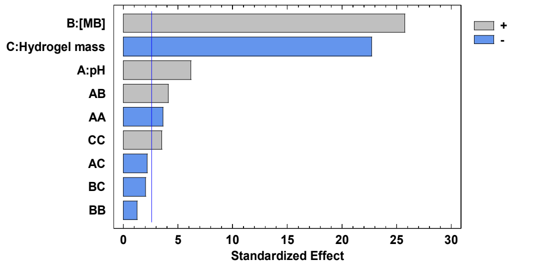

Sample 3: *Thymus lotocephalus, in vitro* cultures, UV-B 1

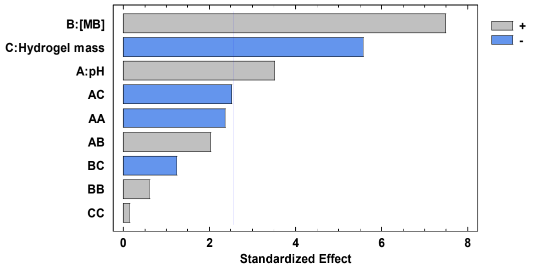

Sample 2: *Lavandula viridis,* micropropagated plants, CT

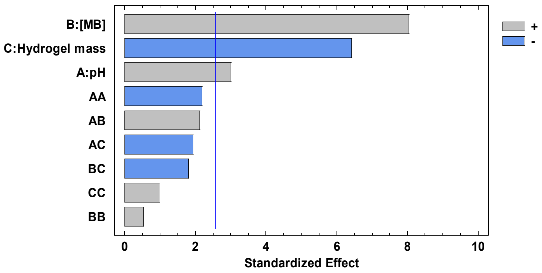

Sample 4: *Thymus lotocephalus,* micropropagated plants, UV-B 4

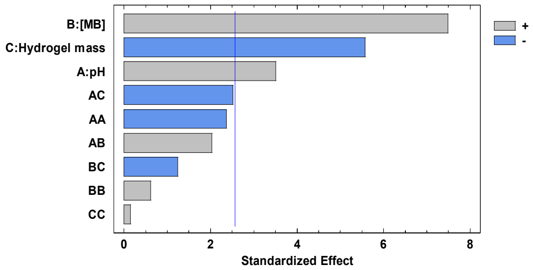

Supplement: Supplementary file 1 [file plants-15-02210-s001.zip › plants-4381746-supplementary.pdf]
